# Steel Based Precious Group Metal‐Free High‐Performance Electrodes for Alkaline Exchange Membrane Water Electrolysis

**DOI:** 10.1002/cssc.70895

**Published:** 2026-07-16

**Authors:** Lukas Heinius, Pierre Schröer, Vincent Wilke, Merel Rittel, Quy Duy Doan, Chen Yie Thum, Raffaele Amitrano, Christian M. Günther, Johannes Schmidt, Duc Van Dinh, Dominik Dworschak, Aldo Saul Gago, Kaspar Andreas Friedrich, Fabio Dionigi, Peter Strasser

**Affiliations:** ^1^ The Electrochemical Energy, Catalysis and Materials Science Laboratory Department of Chemistry Technische Universität Berlin Berlin Germany; ^2^ Institute of Engineering Thermodynamics German Aerospace Center (DLR) Stuttgart Germany; ^3^ Center for Electron Microscopy (ZELMI) Technische Universität Berlin Berlin Germany; ^4^ Department of Chemistry, Functional Materials Technische Universität Berlin Berlin Germany; ^5^ Paul‐Drude Institute for Solid‐State Electronics (PDI) Berlin Germany; ^6^ Helmholtz Institute Erlangen‐Nürnberg for Renewable Energy (IET‐2) Forschungszentrum Jülich Erlangen Germany

**Keywords:** alkaline water electrolysis, chemical engineering, electrode, electrolysis of water, hydrogen production, materials science, overpotential, oxygen evolution

## Abstract

The development of platinum‐group‐metal (PGM)‐free electrodes is essential for cost‐effective alkaline water electrolysis. Herein, we report steel‐based electrodes for a fully PGM‐free, ionomer‐free anion exchange membrane water electrolyzer (AEMWE). A Ni layer deposited on stainless steel (ss) was modified via hydrothermal NiMo growth followed by reductive annealing, yielding a hierarchical NiMo@ss catalyst. Structural characterization revealed MoO_2_‐derived needle‐like structures decorated with metallic Ni and intimate NiMo interfaces. In 0.1 M KOH, the catalyst exhibited an overpotential of −70 mV at −10 mA cm^−2^ with an apparent Tafel slope of 132 mV dec^−1^, demonstrating significantly improved hydrogen evolution reaction kinetics compared to bare stainless steel and electrodeposited Ni. Oxygen evolution reaction (OER) activity of the porous transport layer was enhanced via a simple Ni deposition and anodization procedure, resulting in an overpotential of 284 mV at 10 mA cm^−2^. Integration of both electrodes into a PGM‐free AEMWE single cell (5 × 5 cm^2^) enabled a current density of 1 A cm^−2^ at 1.92 V in 1 M KOH at 60 °C. The cell further demonstrated stable operation for 60 h under dynamic conditions cycling between 0.1 and 1 A cm^−2^. These results highlight the potential of engineered steel‐supported electrodes for scalable noble‐metal‐free hydrogen production.

## Graphical abstract

Steel‐supported, PGM‐free NiMo cathodes and anodized Ni‐coated steel anodes enable an ionomer‐free anion exchange membrane water electrolyzer. The fully noble‐metal‐free single cell delivers 1 A cm^−^^2^ at 1.92 V in 1 M KOH (60 °C) with stable operation over 60 h under dynamic conditions, demonstrating a scalable approach to cost‐effective green hydrogen production.

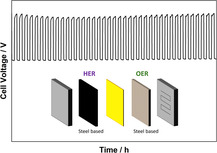

## 1 Introduction

Renewable energy systems are essential to mitigate rising CO_2_ emissions but require efficient storage and conversion technologies to balance fluctuating supply and demand [1]. Green hydrogen, produced via water electrolysis using renewable electricity, represents a key energy vector enabling long‐term storage and sector coupling. Beyond its direct use in fuel cells or gas turbines for electricity generation, hydrogen serves as a feedstock for large‐scale chemical processes such as ammonia and methanol synthesis [2].

Indeed, the global demand for hydrogen has risen steadily over the last decade, with a growth of 2% from 2023 to 2024 alone [3]. This demand is largely met by fossil fuels, while production from renewable energy sources has increased in kind, but still only accounts for 10% of total hydrogen production. Despite the growing interest in low‐emissions hydrogen, the sector has not yet met the ambitious targets set in recent years. High production costs, uncertain demand, regulatory challenges, and slow infrastructure development have all contributed to this gap. However, there are still notable signs of growth. For example, although final investment decisions (FIDs) continue to trail well behind announcements, over 200 low‐emissions hydrogen production projects have received FIDs since 2020, compared to just a handful of demonstration projects in operation. Innovation is advancing rapidly, with a record number of technologies across the hydrogen value chain showing significant progress over the past year.

One bottleneck in emerging water electrolysis technologies is the reliance on critical raw materials like platinum and iridium for proton exchange membrane (PEM) electrolyzers [4]. Among water electrolysis technologies, alkaline exchange membrane water electrolysis (AEMWE) has emerged as a promising alternative to conventional alkaline electrolysis (AEL) and PEM water electrolysis [5]. AEMWE aims to combine the advantages of both systems: the use of nonprecious metal catalysts and cost‐effective cell components typical for AEL, with the compact design and high current densities characteristic of PEMWE [6]. In particular, the ability to employ platinum‐group‐metal (PGM)‐free catalysts significantly reduces material costs and critical raw material dependency.

Furthermore, coupling AEM electrolyzers to renewable energy sources introduces dynamic operating conditions with frequent load changes. Such fluctuations impose additional mechanical and electrochemical stress on catalyst layers and substrates, potentially accelerating degradation processes, including metal dissolution, surface reconstruction, and delamination [7]. Nickel‐based catalysts, especially NiMo alloys, are widely investigated for the hydrogen evolution reaction (HER) in alkaline media due to their high intrinsic activity and favorable hydrogen binding energy. However, their long‐term stability remains a critical challenge, particularly under industrially relevant current densities [8, 9]. Possible deactivation mechanisms include the leaching of molybdate (MoO_4_^2−^) ions, severe reconstruction, and surface roughening during electrocatalysis—especially under variable load operation, where oxidative stress is applied to the cathode during shutdown [10].

Stainless steel (ss) substrates offer an attractive platform for cost‐efficient electrode fabrication due to their mechanical robustness, electrical conductivity, and scalability [11, 14]. Nevertheless, corrosion phenomena and interfacial instability under highly alkaline and dynamic conditions remain insufficiently understood [15].

To address these challenges, accelerated stress tests (ASTs) simulating dynamic operation conditions are required. In this work, load cycling between 1 and 0.1 A cm^−2^ was employed to mimic fluctuating renewable energy input, as well as high‐stress environments, especially for the molybdenum‐containing cathode, while minimizing molybdenum leaching by avoiding open circuit voltage (OCV) [16]. While electrolyzer tests have been conducted with nickel‐molybdenum as the cathode, these studies were mostly limited to either small single‐cell areas or did not involve variable load operation [17, 18].

Herein, we report the development of a high‐performance, stable steel‐supported NiMo catalyst for the cathode in AEM water electrolysis. NiMo was deposited onto ss via a three‐step synthesis procedure, yielding a mechanically robust and highly active electrode architecture. The catalyst performance and degradation behavior were systematically evaluated under dynamic load conditions using aAST. This approach provides insight into the stability limitations of NiMo‐based electrodes and demonstrates a viable strategy for cost‐efficient and durable AEMWE systems.

## 2 Experimental Section

### 2.1 Synthesis

The stainless‐steel felts (dioxide materials GDL ss fiber paper) and meshes (Goodfellow AISI 316 stainless steel mesh) were laser cut to a size of 5 × 5 cm^2^. The PTLs were then cleaned by sonicating them in 3 M HCl for 10 min followed by rinsing them with Milli‐Q H_2_O (18.2 MOhm cm) and isopropanol. To increase the corrosion resistance of the meshes, a nickel layer of 5 mg cm^−2^ was introduced by electrodeposition (ED) from a Ni‐watts bath.

### 2.2 Synthesis of Anode Catalyst: Anodized Ni on Stainless Steel

The synthesis of the anode is based on a protocol suggested by Ekspong et al., however, with significant modifications [16].

A cathodic degreasing protocol in a bath containing 60 g L^−1^ of JE610 (Jentner) electrolytic degreasing salt with Ni plate anodes was carried out for 60 s at 6 V. Then the felts were rinsed with milQ water, pickled for 15 s in 3 M HCl and rinsed again with milQ water. To increase the outcome of the ED an electroless nickel plating bath was prepared using NiSO_4_*6H_2_O (20 g L^−1^, Roth, >98%) as the nickel source and sodium hypophosphite (20 g L^−1^) as the reducing agent. Maleic acid (20 g L^−1^) and sodium succinate (20 g L^−1^) were added as complexing agents. Pb(OAc)_2_ (1 mg L^−1^) and sodium thiosulfate (1 mg L^−1^) were introduced as stabilizing agents. The bath was heated to 85 °C and under continuous stirring the substrate was immersed in the solution for 30 min to achieve nickel deposition. After plating, the samples were removed and rinsed with milQ H_2_O and isopropanol. The cleaned and dried felts were weighed and then placed in a Ni‐Watts bath containing 30 gL^−1^ NiCl_2_*6H_2_O (thermoscientific, 99%, 240 g L^−1^ NiSO_4_*6H_2_O (Roth, >98%) and 30 g L^−1^ H_3_BO_3_ (VWR, 100%). Nickel plates were used as anodes and the current was set to 0,04 A cm^−2^ = 1 A at 5 × 5 cm^2^ for a duration of 14 min, leading to a Ni loading of 4.5 mg cm^−2^, which equates to a layer thickness of roughly 5 µm (see S19). Lastly, an anodization step in 7.5 M NaOH for 5 min at 5 V with a stainless‐steel counter electrode was carried out.

### 2.3 Synthesis of Cathode Catalyst: NiMo on Ni‐Plated Steel

A cathodic degreasing protocol in a bath containing 60 g L^−1^ of JE610 (Jentner) electrolytic degreasing salt with Ni plate anodes was carried out for 60 s at 6 V. Then the felts were rinsed with milQ water, pickled for 15 s in 3 M HCl and rinsed again with milQ water. To increase the outcome of the ED an electroless nickel plating bath was prepared using NiSO_4_*·6H_2_O (20 g L^−1^, Roth, >98%) as the nickel source and sodium hypophosphite (20 g L^−1^) as the reducing agent. Maleic acid (20 g L^−1^) and sodium succinate (20 g L^−1^) were added as complexing agents. Pb(OAc)_2_ (1 mg L^−1^) and sodium thiosulfate (1 mg L^−1^) were introduced as stabilizing agents. The bath was heated to 85 °C and under continuous stirring, the substrate was immersed in the solution for 30 min to achieve nickel deposition. After plating, the samples were removed and rinsed with milQ H_2_O and isopropanol. The cleaned and dried felts were weighed and then placed in a Ni‐Watts bath containing 30 g L^−1^ NiCl_2_*6H_2_O (thermoscientific, 99%, 240 g L^−1^ NiSO_4_*6H_2_O (Roth, >98%) and 30 g L^−1^ H_3_BO_3_ (VWR, 100%). Nickel plates were used as anodes and the current was set to 0,04 A cm^−2^ = 1 A at 5 × 5 cm^2^ for a duration of 80 s leading to a Ni loading of 0.45 mg cm^−2^, which equates to a layer thickness of roughly 0.5 µm (see S19). The thin Ni layer was deposited to increase the affinity of NiMo to the surface of the steel substrate. A solution of 500 mL containing 0,02 mol Ni(NO_3_)_2_*6 H_2_O and (Roth, >99%) and 0,02 mol NH_4_MoO_7_*4 H_2_O (Sigma Aldrich, >99%) was prepared. The sample was set flat on a holder, and the precursor solution was slowly filled into the microwave vial. The hydrothermal reaction was carried out in an Anton par Masterwave. The solution was heated to 160 °C, held for 60 min and then slowly cooled to room temperature. The success of the reaction was shown by a yellow deposit on the substrate surface (Figure S1). The felt was carefully lifted out of the microwave vial, dried at 60 °C overnight and then transferred to a tube furnace. In the last synthesis step, the catalyst was heated at a ramp of 5 K min^−1^ and then reduced for 1 h at 550 °C in 4% H_2_‐containing Ar gas and slowly cooled to room temperature. The resulting catalyst showed a thin black layer on its metallic surface (Figure S2).

### 2.4 X‐Ray Diffraction (XRD) Measurements

XRD patterns were measured using a Bruker D8 Advance X‐ray powder diffractometer equipped with a Lynx Eye detector and a Cu Kα radiation source. The diffractogram was recorded in step scan mode with a scanning rate of 0.24°/s and a step size of 0.12° in a 2θ range from 10° to 80°. The samples were placed in a cavity holder, ensuring precise focus of the X‐ray beam.

### 2.5 Scanning Electron Microscopy (SEM)

High‐resolution imaging was performed using a ZEISS Gemini 500 NanoVP field emission SEM (FE‐SEM) equipped with a Gemini I column and a Bruker Quantax XFlash 6|60 EDX detector. The system provides a spatial resolution of up to 0.6 nm at 15 kV and 1.2 nm at 500 V using the Nano‐Twin lens configuration. For compositional analysis, energy‐dispersive X‐ray spectroscopy (EDX) was conducted at an accelerating voltage of 15 kV and a working distance of 8.5 mm.

### 2.6 X‐Ray Photoelectron Spectroscopy (XPS)

XPS measurements were acquired using Al K_α_ + X‐ray photoelectron spectrometer system (Thermo Scientific). The instrument features a hemispheric 180° dual‐focus analyzer with a 128‐channel detector. Energy steps of 1.0 and 0.1 eV were used for survey and high‐resolution measurements, respectively. A spot size of 400 µm was used. The C 1s binding energy of 284.4 eV was used for calibration.

### 2.7 Focused Ion Beam (FIB)

FIB preparation of the samples was conducted at the nano‐workbench at ZELMI using a FEI/ThermoFisher Helios Nanolab 600 Dualbeam. After deposition of a Pt protective layer, cross‐sections of 20 µm in width were milled using Ga‐ions at 30 kV with currents of 2.8 and 0.28 nA. SEM‐imaging was performed in UHR mode at 5 kV at a working distance of 3 mm.

### 2.8 Inductively Coupled Plasma Mass Spectrometry (ICP‐MS)

The anolyte, catholyte, and pure electrolyte samples were diluted by a factor of 100 and acidified with 3% HNO_3_. A standard series was created for Ni, Fe, and Mo with the following concentrations: 0.01, 0.05, 0.2, 0.5, 1.0, and 5.0 mg L^−1^. ICP‐MS measurements were performed at an Agilent 7900 (Agilent Technologies) in He mode (5 mL min^−1^), and fourfold dilution with HMI. 10 µg L^−1^ Rh was used as an internal standard for ^95^Mo.

### 2.9 Three Electrode Setup Measurements

For the small‐scale evaluation of electrocatalytic activity, 2 × 1 cm^2^ electrodes were prepared. The 1 × 2 cm^2^ electrodes used for the HER and OER three‐electrode performance test were synthesized almost analogous to the 5 × 5 cm^2^ electrodes used for AEMWE. The ED step was adapted to the smaller area, keeping the loading at 0.45 and 4.5 mg cm^−2^, respectively. The hydrothermal reaction used for the NiMo HER catalyst was carried out in a smaller Anton Paar Monowave with a volume of 15 mL keeping the reaction conditions analogous to the 5 × 5 cm^2^ electrodes.

The 2 × 1 cm^2^ electrodes were placed in a L‐shaped holder contacting the steel felt with screws to the electrical interface using a Biologic SP‐200 potentiostat and thus reducing the electrochemically active area to 0.75 cm^2^. The performance was tested in N_2_‐saturated 0.1 M KOH solution in a glass beaker‐type cell. A reversible hydrogen electrode (RHE) by Gaskatel was used as the reference electrode. A high surface Ni felt was used as the counter electrode.

The HER protocol consists of eight steps: chronoamperometry (CA) (1), linear sweep voltammetry (LSV) (2), potentiostatic electrochemical impedance spectroscopy (PEIS) (3), LSV (4), cyclic voltammetry (CV) (5), LSV (6), PEIS (7), and CV (8).

The OER protocol consists of eight steps: OCV (1), CA (2), PEIS (3), CV (4), LSV (5), PEIS (6), CA (7), and PEIS (8).

A 100% iR compensation was applied, resulting in the iR corrected LSV curves. The Tafel slopes were calculated in the low current part of the LSV. The Tafel slopes in this study should be considered “apparent” Tafel slopes, due to the possible contribution of mass transport phenomena in porous electrodes used in this study.

To determine the adsorption capacitance (*C*_a_), staircase PEIS (SPEIS) was employed following a procedure adapted from Jeon et al. Rather than recording a single PEIS spectrum at one potential, impedance spectra were acquired over a range of applied potentials to identify the most suitable potential window for capacitance determination.

For HER measurements, impedance spectra were recorded between −0.7 and 0.0 V versus RHE, whereas for OER measurements the potential range was 1.2–1.7 V versus RHE. In both cases, the potential was increased in 10 mV increments. At each potential, impedance spectra were collected over a frequency range from 18 kHz to 1 Hz using an AC perturbation amplitude of 10 mV.

The resulting impedance spectra were analyzed to extract the adsorption capacitance values (*C*_a_). Representative spectra and the complete dataset are provided in the Supporting Information (Figures S32–S39).

### 2.10 AEMWE Cell Measurements

The AEMWE single‐cell measurements were carried out in an electrolyzer testing station (Bürkle EEZT‐AEM) in 1 M KOH at a temperature of 60 °C. An AF3‐HWC9‐70‐0A4‐0005‐X Aemion+ reinforced anion exchange membrane by Ionomr was used. Activation was done by immersing the membrane in 1 M KCl for 24 h and in 1 M KOH for 100 h. The anolyte flow was set to 100 mL min^−1^ while the cathode was kept dry. A sealing thickness of 900 µm and a torque of 5 N m were used for the compression of the cell. The loading of the cathode was determined by weight and was equal to 3 mg cm^−2^. Goodfellow AISI 316 stainless steel meshes with a thickness of 0.45 mm were used to optimize mass transport and the tightness of the cell. Corrosion resistance and conductivity were increased by depositing a 5 μm thick Ni layer on the mesh surface.

The following protocol was used for the AEMWE single‐cell measurement: an activation phase consisting of a 20 h hold at 1 A cm^−2^, a polarization curve, AST cycling between 1 and 0.1 A cm^−2^ for more than 20 h, which was followed by a constant current hold at 1 A cm^−2^ for an additional 12 h. A data point was recorded every second of the measurement. The polarization curve was measured between 0.01 and 1 A cm^−2^, with a scan rate of 0.3 mA cm^−2^ s^−1^.

The current limits for the AST were applied for 15 min, resulting in a 30 min full cycle. A total number of 40 cycles were performed.

## 3 Results and Discussion

**FIGURE 1:**
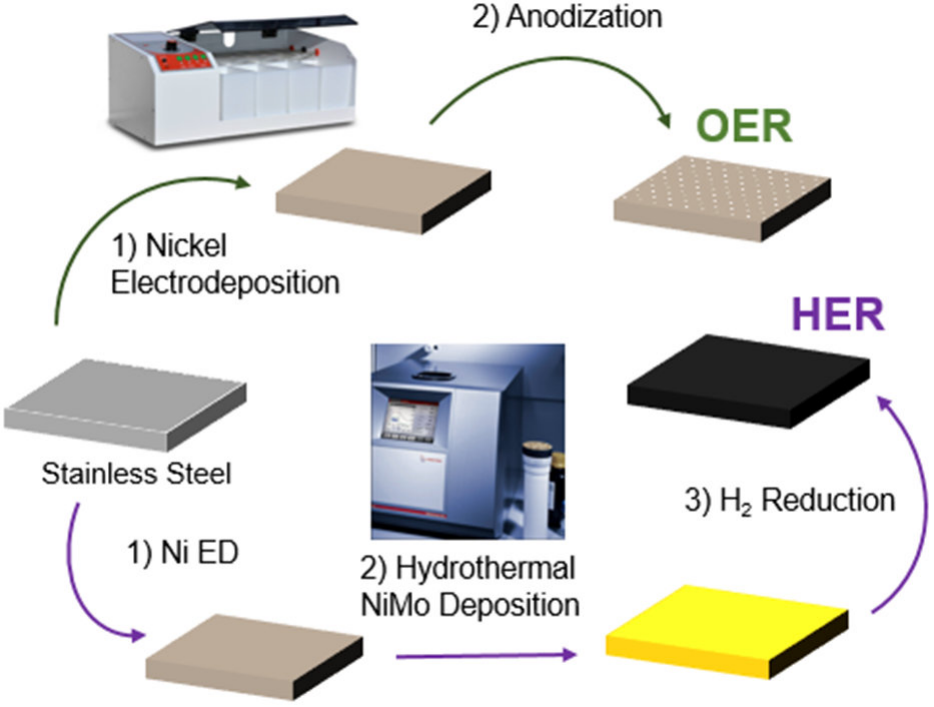
Synthesis scheme of the stainless steel‐based OER and HER catalysts. The OER catalyst is synthesized starting from stainless steel in a two‐step process consisting of nickel electrodeposition (ED) and a subsequent surface anodization. The HER catalyst is produced in three‐steps: (1) nickel ED, (2) hydrothermal NiMo deposition, and (3) thermal H_2_ reduction (Figures S1,S2).

In this study, we present two stainless steel‐based electrodes for the application in PGM‐free AEMWE. A three‐step synthesis procedure was used to produce NiMo on stainless steel as a CCS‐type cathode catalyst. The general approach was inspired by literature, and then iteratively improved to increase performance and stability in the electrolyzer flow cell [19]. The process is shown in Figure 1. In the first step, a thin Ni layer was established using a Ni ED bath and a constant current. This thin Ni layer allowed a homogenous deposition of NiMo using a hydrothermal microwave‐assisted method [20, 21]. In the last step, a thermal reduction was carried out to produce the final NiMo@ss HER catalyst. The anode catalyst was synthesized by electrodepositing a thin Ni layer from a Ni‐Watts bath onto the stainless‐steel surface. This Ni‐enriched surface was then anodized in an alkaline bath to increase the electrocatalytic performance (see Figure 1) [22].

### 3.1 Physicochemical Characterization

To investigate the structure and elemental composition of the catalyst materials, various physicochemical characterization methods such as SEM with energy dispersive detector (EDX), FIB‐SEM, XRD, and XPS were carried out (see Figures S5–S8, S11−S14, S17−S21, S24−S25 and S28−S32).

### 3.2 NiMo@ss (Ni@MoO_2_ on Porous Transport Layer)

**FIGURE 2:**
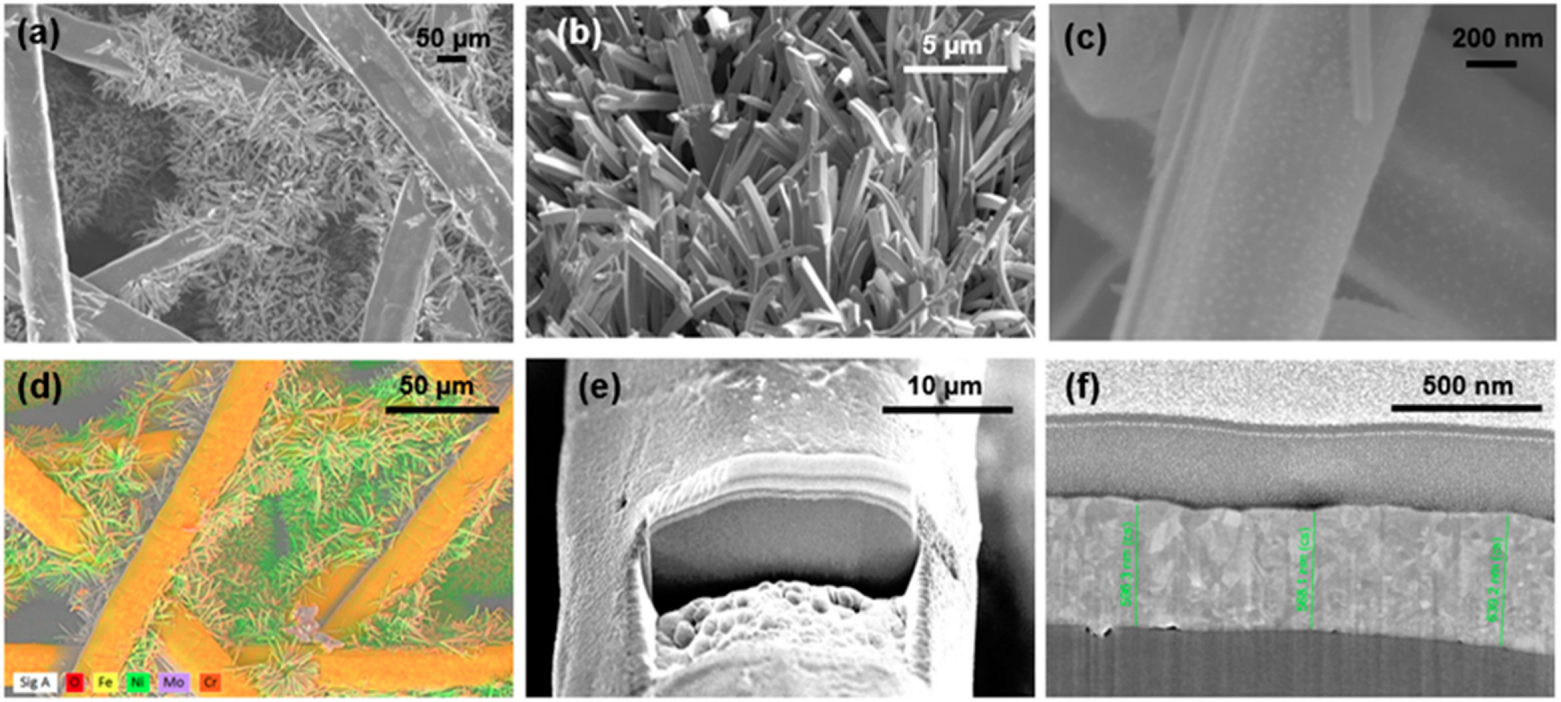
Physicochemical characterization of the stainless‐steel‐based electrodes to provide insight into the morphology and layer properties. (a–d) SEM images of NiMo@ss with (d) EDX mapping and (e,f) SEM images of a FIB cross‐section of anodized Ni@ss.

SEM images of NiMo@ss reveal a wire‐like microstructure of the porous transport layer (PTL) decorated with multiple micrometer‐long needle‐like features (Figure 2a,b). These structures consist of MoO_2_ with Ni nanoparticles distributed across their surface, resulting in pronounced surface roughening and hierarchical morphology (Figure 2c).

The H_2_ annealing preserved the one‐dimensional rod‐like morphology while increasing crystallinity and promoting metallic Ni formation. The improved electronic conductivity and strengthened NiMo interfacial interaction are expected to enhance HER kinetics.

EDX mapping confirms the presence of both Mo and Ni uniformly distributed along the wire‐like framework of the PTL, verifying the successful deposition of Ni on MoO_2_. The intimate contact between Ni nanoparticles and the conductive MoO_2_ backbone is expected to facilitate charge transfer and create synergistic catalytic interfaces (Figure 2d).

The combination of a high‐aspect‐ratio needle‐like morphology, and homogeneous nickel dispersion over the Mo monoliths, likely enhances the electrochemically active surface area (ECSA) and improves mass transport, thereby contributing to the improved HER performance, without impeding the ability of the PTL to efficiently remove formed hydrogen at the cathode.

### 3.3 Ni/Ni(OH)_2_/NiO Layer on Stainless Steel

SEM analysis reveals a compact and homogeneous coating on the stainless‐steel substrate after Ni deposition (Figure 2a,b). EDX confirms that the surface is predominantly composed of Ni (90.6 at%), while minor contributions from Fe (6.4 at%) and Cr (1.4 at%) originate from the underlying stainless‐steel substrate.

Upon anodization, the oxygen content increases from negligible values to approximately 1.6 at%, indicating the formation of a thin NiO layer on the surface. This compositional change confirms the successful partial oxidation of Ni and the generation of a Ni/NiO /Ni(OH)_2_ interface.

The thickness of the deposited Ni/NiO/Ni(OH)_2_ layer was determined by FIB‐SEM cross‐sectional analysis to be 0.59 ± 0.04 µm, demonstrating controlled and reproducible film growth under the selected deposition conditions (Figure 2e,f). The formation of a thin oxide layer is expected to increase surface roughness and therefore oxygen evolution activity.

**FIGURE 3:**
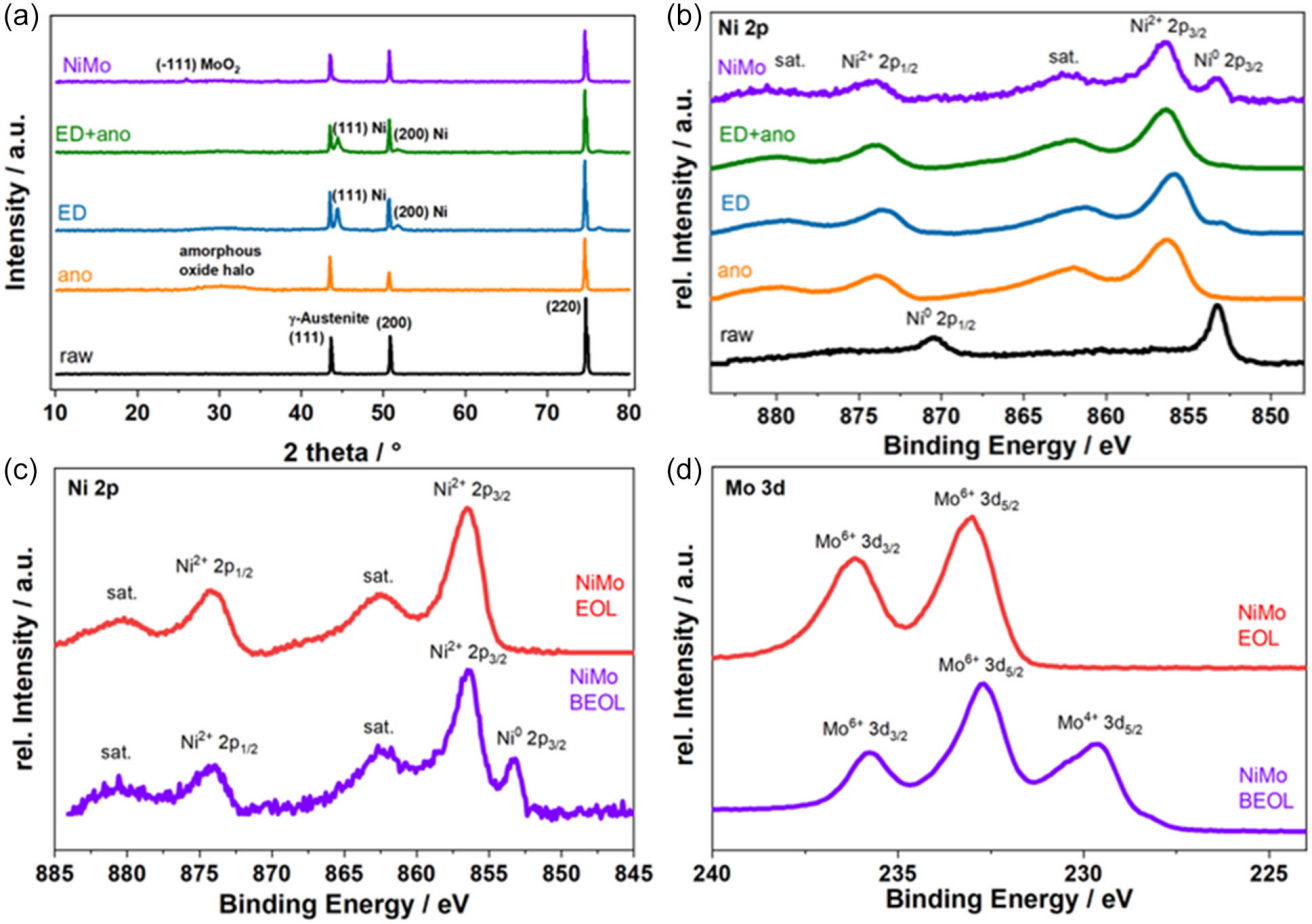
(a) X‐ray diffractograms of raw steel (black), anodized steel (orange), Ni electrodeposition (ED, blue), Ni electrodeposition with a subsequent anodization step (green) and NiMo@ss (purple); (b) 2p XPS spectra of the mentioned materials; (c) Ni 2p XPS of NiMo@ss before cell test (BEOL) (purple) and after cell test (EOL) (red); and (d) Mo 3d XPS‐spectra of NiMo@ss BEOL (purple) and EOL (red).

In addition to SEM/FIB SEM imaging, XRD, and XPS were used to gain deeper insight into the formation of the active components on the PTLs (see Figure 3).

### 3.4 XRD Analysis

XRD was employed to characterize the structural features of the samples and verify the success of the anodization and deposition processes. The XRD patterns of all samples (Figure 3a) exhibit typical steel characteristics, notably the prominent peaks associated with the face‐centered cubic (fcc) γ‐austenite (γ‐Fe) crystal structure of the substrate.

The XRD peaks at 43.6° (111), 50.8° (200), and 74.7° (220) are clearly visible in all samples, corresponding to the characteristic reflections of the fcc γ‐austenite phase (γ‐Fe) of the steel substrate [23]. These peaks are indicative of the presence of a well‐ordered crystalline structure typical of steel, confirming the integrity of the substrate prior to any further processing steps.

The success of the anodization process was confirmed by the appearance of an amorphous oxide halo in the XRD pattern, observed in the 25° to 35° 2θ range. This halo is attributed to the formation of amorphous iron oxide and chromium oxide layers, which are generated by the strong anodic potential applied during the anodization process under alkaline conditions. The broad, noncrystalline halo further supports the formation of a thin, noncrystalline oxide layer, which is consistent with previous reports on anodization of stainless steel in such environments, with a shift to lower Bragg angles, indicating an oxide and carbon mixture in the matrix [24].

Upon deposition of nickel (Ni) onto the anodized steel surface, distinct XRD reflections corresponding to the crystalline structure of nickel emerge. While the most prominent Bragg reflections are still the austenitic steel, pronounced reflections are also observed at 45.4° and 51.8°, which correspond to the (111) and (200) crystal planes of the fcc Ni lattice [25]. These reflections confirm the successful deposition of metallic nickel and its crystalline nature on the substrate. The presence of these peaks further suggests that the deposition process was well‐executed, leading to the formation of a high‐quality metallic Ni layer on the surface. Even upon Anodization at 5V in 7.5 M KOH, the metallic nickel reflections do not disappear, implying passivation with a nickel oxide/hydroxide interface not discernible via XRD.

The subsequent hydrothermal reaction and reduction process of the NiMo@ss HER catalyst give rise to the most intense diffraction peak corresponding to the (−111) plane of MoO_2_. This reflection, typically observed at a 2θ value of 26.0, is the most pronounced in the XRD pattern area between 20° and 30° and is indicative of the successful formation of the MoO_2_ phase, which is crucial for the catalytic activity in HER.

However, it is worth noting that the additional reflections from MoO_2_ cannot be resolved due to the polycrystalline nature of the material. The overlapping diffractions from the different crystallographic planes of MoO_2_, along with the possible presence of other phases, contribute to this broadening, preventing a clear distinction of individual reflections beyond the (−111) peak. This is a common phenomenon when dealing with polycrystalline materials that exhibit lower crystallinity, and further analysis, such as high‐resolution XRD or other characterization techniques, may be required to resolve finer details of the MoO_2_ phase.

### 3.5 X‐Ray Photoelectron Spectroscopy

XPS was employed to analyze the near‐surface chemical composition of the modified stainless‐steel electrodes. With a typical information depth of approximately 5–10 nm, XPS is well‐suited to probe chemical changes occurring at the catalyst–electrolyte interface.

Since all catalyst systems investigated in this study involve nickel‐containing surface modifications, particular attention was given to the Ni 2p core level region (Figure 3b). The untreated stainless‐steel substrate exhibits a pronounced Ni 2p_3/2_ peak at 852.7 eV, characteristic of metallic Ni species typically present in austenitic stainless steel [26] (see Figures S9,S10).

Following anodic treatment, the Ni 2p core levels shift toward higher binding energies, accompanied by the emergence of satellite features characteristic of oxidized nickel species [24]. This observation indicates the formation of Ni^2+^‐containing surface species, consistent with nickel oxide or hydroxide formation. Deposition of an additional nickel layer results in the coexistence of metallic and oxidic nickel species at the surface, suggesting partial oxidation during or after deposition. Subsequent anodization of the nickel‐coated substrate leads to similar spectral features, indicating that surface oxidation dominates regardless of whether the substrate is directly anodized or first nickel‐coated [27] (see Figures S15,S16,S22,S23, S26,S27).

Upon deposition of NiMoO_*x*_ and subsequent reductive activation, both metallic and oxidized nickel species are detected. The persistence of oxidized nickel after reductive treatment is not unexpected, as nickel readily forms a passivating oxide layer upon air exposure, unless handled under strictly inert conditions [28] (see Figures S34,35).

The Mo 3d region reveals the presence of molybdenum in mixed oxidation states, predominantly Mo^4+^ and Mo^6+^, consistent with partially oxidized molybdenum species at the surface [29] (Figure S33).

Significant spectral changes are observed after prolonged electrochemical cell testing under constant and varying current (Figure 3c). The metallic Ni component at roughly 853 eV is no longer detectable within the signal‐to‐noise limits, while the Ni 2p spectrum is dominated by oxidized species. Concurrently, the Mo 3d signal in the binding energy range associated with Mo^4+^ diminishes, with features consistent with Mo^6+^ species becoming predominant. These observations suggest substantial surface oxidation during electrochemical operation (see Figures S36,37).

Although the electrochemical protocol was designed to minimize oxidative degradation, exposure to AEL and dynamic polarization likely promotes the formation of nickel hydroxides and oxidative transformation of molybdenum species. Dissolution and redeposition processes of molybdenum under alkaline conditions may further contribute to the observed changes. In the anolyte, a Mo concentration of 52.9 ± 1.4 mg L^−1^ was determined by ICP‐MS (see Table S1). This is roughly a dissolution of about 10% of the total Mo incorporated in the electrode.

Notably, despite these surface transformations, the electrode maintains its catalytic activity during extended cycling. This suggests the formation of a stable, catalytically active NiMoO_*x*_‐derived surface phase under operating conditions. The morphological changes observed by SEM are consistent with this restructuring process (see Figures 2, S28–S32 and S38,S39) [30].

### 3.6 Electrochemical Analysis and Performance

To investigate the electrochemical activity on a small scale and to screen materials for AEMWE single‐cell measurements, three‐electrode measurements were carried out in 0.1 M KOH in a beaker‐type cell. The 2x1 cm samples were measured against a high surface area nickel mesh and a RHE was used as the reference electrode.

### 3.7 HER Performance

**FIGURE 4:**
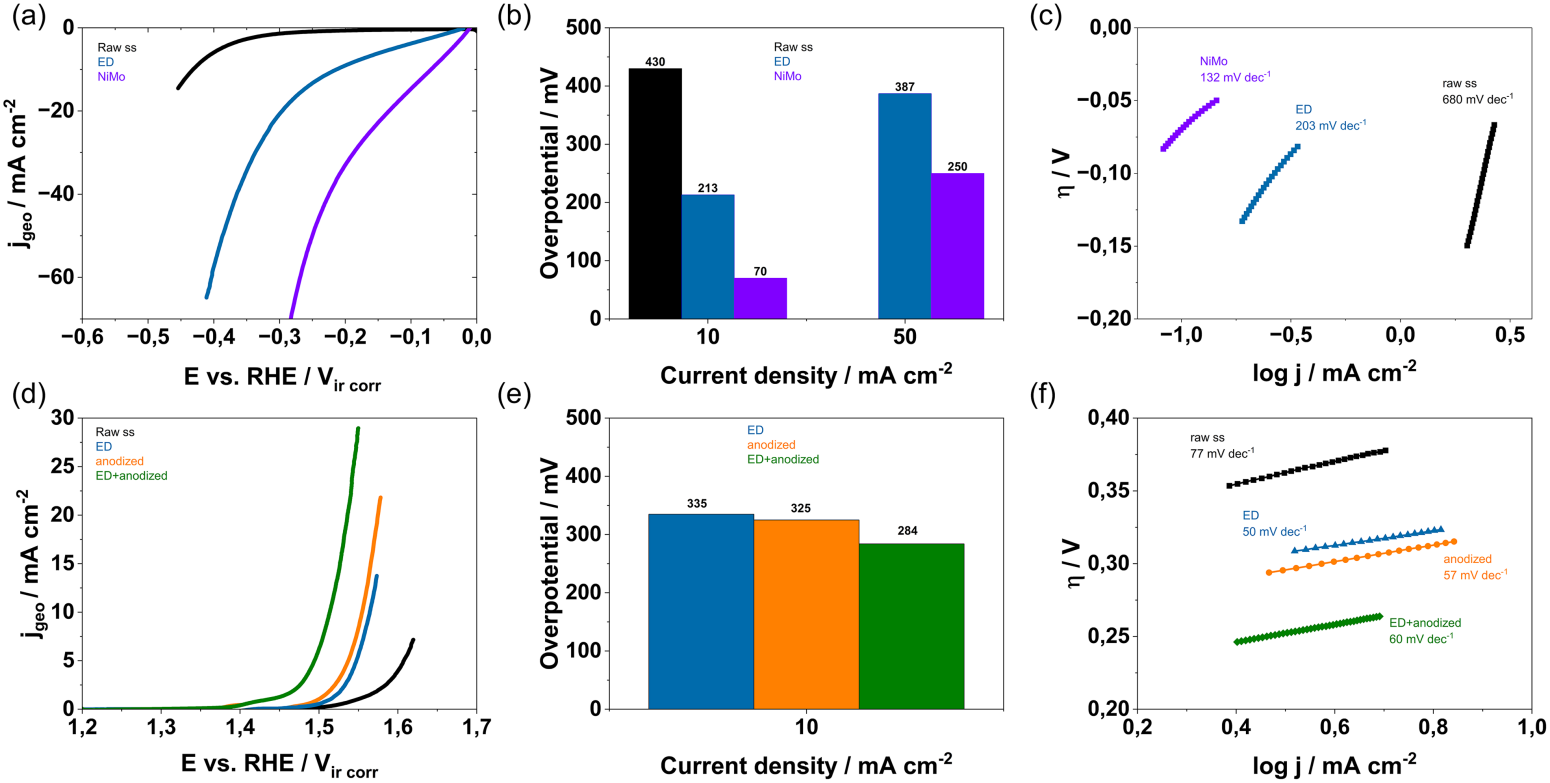
Electrochemical performance evaluation using a small‐scale three electrode setup (a) LSV HER curves of raw stainless steel (black), Ni electrodeposited—ED (blue), NiMo@ss (purple); (b) HER overpotentials at −10 and −50 mA cm^‒^^2^ compared; (c) Tafel slopes; (d) LSV OER curves for raw stainless steel (black), ED (blue), anodized steel (orange), ED + anodized steel (green); and (e) overpotentials at 10 mA cm^‒^^2^ compared; (f) Tafel slopes.

Raw stainless‐steel substrate had the HER lowest performance with an overpotential of −430 mV at −10 mA cm^−2^. Upon ED of a Nickel layer the overpotential shrinks to −213 mV at −10 mA cm^−2^ and −387 mV at −50 mA cm^−2^ (see Figure 4a,b, blue line). Using a hydrothermal NiMo deposition and a subsequent thermal reduction step resulted in the NiMo@ss HER catalyst and lowered the overpotential to −70 mV at −10 mA cm^−2^ and −250 mV at −50 mA cm^−2^ (Figure 4a,b, purple line).

The HER activity of the hydrothermally synthesized NiMo@ss catalyst compares favorably with previously reported NiMo systems, despite the relatively low surface area of stainless‐steel substrates and the mild 0.1 M KOH electrolyte. According to literature, Ni–Mo alloys supported on high‐surface‐area Ni foam typically achieve overpotentials as low as −30 to −42 mV at −10 mA cm^−2^ in 1 M KOH, benefiting from both increased geometric surface area and enhanced charge transport. Vertical graphene‐supported NiMo and NiMoO_4_ nanorod‐based electrodes exhibit overpotentials of −71 to −127 mV at 10 mA cm^−2^, while NiMo nanoparticles on Ti substrates reach roughly −252 mV at −10 mA cm^−2^ [21, 31, 33]. In this context, the NiMo@ss catalyst delivers an overpotential of −70 mV at −10 mA cm^−2^. Comparable Ni–Mo catalysts tested in 0.1 M KOH show overpotentials in the range of −150 to −200 mV at −10 mA cm^−2^ in RDE measurements, indicating that the −70 mV overpotential observed for NiMo@ss is significantly better than many unsupported NiMo materials under similar electrolyte conditions [20, 34]. The superior performance can be attributed to the hierarchical needle‐like MoO_2_ rods decorated with Ni nanoparticles, which provide abundant heterointerfaces, high ECSA, and efficient electronic pathways. These results highlight the synergistic interaction between Ni and Mo, enhancing the Volmer step and overall HER kinetics. Overall, the combination of robust substrate anchoring, hierarchical morphology, and Ni–Mo electronic synergy allows NiMo@ss to achieve competitive HER performance relative to state‐of‐the‐art Ni–Mo catalysts under both mild and industrially relevant alkaline conditions.

To further evaluate the influence of the catalyst morphology on the accessible surface area, adsorption capacitance measurements were performed using an impedance‐based approach adapted from Jeon et al. The extracted capacitance values are summarized in Table 1. For the HER catalyst, the capacitance increased from 2800 µF for bare stainless steel to 70,666 µF for NiMo@ss, corresponding to an approximately 25‐fold increase in electrochemically accessible surface area (S40−S44). Likewise, the electrodeposited Ni and anodized OER electrode exhibited a capacitance of 90,000 µF compared to 17,333 µF for bare stainless steel, corresponding to a 5.2‐fold increase (S45–S48. These results confirm that the applied surface modifications substantially increase the electrochemically accessible surface area and support the enhanced catalytic activity observed in the polarization measurements.

**TABLE 1:** Overview of the extracted adsorption capacitance value *C*_a_ determined with the method adapted from Jeon et al. [35].

Reaction	Substrate	**Capacitance** ***C***_**a**_ **, µF**	Chosen voltage range	Surface enhancement
HER	Stainless steel	2800	0.3−0.5 V	1
HER	NiMo@ss	70,666	0.4–0.6 V	25x
OER	Stainless steel	17,333	1.5–1.6	1
OER	Ed + anodized steel	90,000	1.5–1.6	5.2x

The Tafel slopes were determined in the low current density part of the LSVs in Figure 4a,c. The raw stainless steel showed a large apparent Tafel slope of 680 mV dec^−1^, which is in agreement with the poor intrinsic activity and sluggish kinetics. ED of a thin nickel layer decreased the slope to a value of 203 mV dec^−1^ showing a large performance gain compared to plain stainless steel. NiMo on stainless steel (purple, Figure 4c) has the lowest Tafel slope of 132 mV dec^−1^. This is close to the slope of the Volmer step of roughly 120 mV dec^−1^ and implies Volmer kinetics. This highlights the synergistic effect between Ni and Mo, which improves the kinetics significantly.

### 3.8 Oxygen Evolution Reaction (OER) Performance

Raw stainless steel (black) showed the lowest OER performance with an overpotential of more than 400 mV while only reaching a current density of 7 mA cm^−2^. The activity could be increased by electrodepositing a nickel layer onto the surface, resulting in a decreased overpotential of 335 mV at 10 mA cm^−2^ (Figure 4d,e, blue). The anodized steel sample (orange) had a slightly higher OER activity with an overpotential of 325 mV at 10 mA cm^2^ (Figure 4d,e). The best performing material was the electrodeposited and anodized stainless steel with an overpotential of 284 mV at 10 mA cm^2^ (Figure 4, green).

The Tafel slopes were determined in the kinetically controlled area from the LSVs in Figure 4d and are shown in Figure 4f. Raw stainless steel showed yet again the largest Tafel slope with a value of 77 mV dec^−1^ manifesting its low electrochemical activity. The Ni ED sample (blue) has a Tafel slope of 50 mV dec^−1^, which displays an improved performance. Controlled anodization of the steel surface lowered the Tafel slope compared to raw steel to 57 mV dec^−1^, showing an increased activity due to a potential surface roughening. The combination of both surface modifications resulted in a Tafel slope of 60 mV dec^−1^. Although the electrodeposited and anodized material (green) showed a slightly steeper Tafel slope than the other two materials, it had the lowest overpotential and largest current density in the LSV (see Figure 4d,e) and was thus chosen as the best performing material for the OER electrode.

The two materials with the highest activity in HER (NiMo, purple) and OER (ED + ano, green) were chosen for AEMWE single‐cell measurements at an increased electrode size of 5 × 5 cm^2^.

### 3.9 AEMWE Single‐Cell Test

**FIGURE 5:**
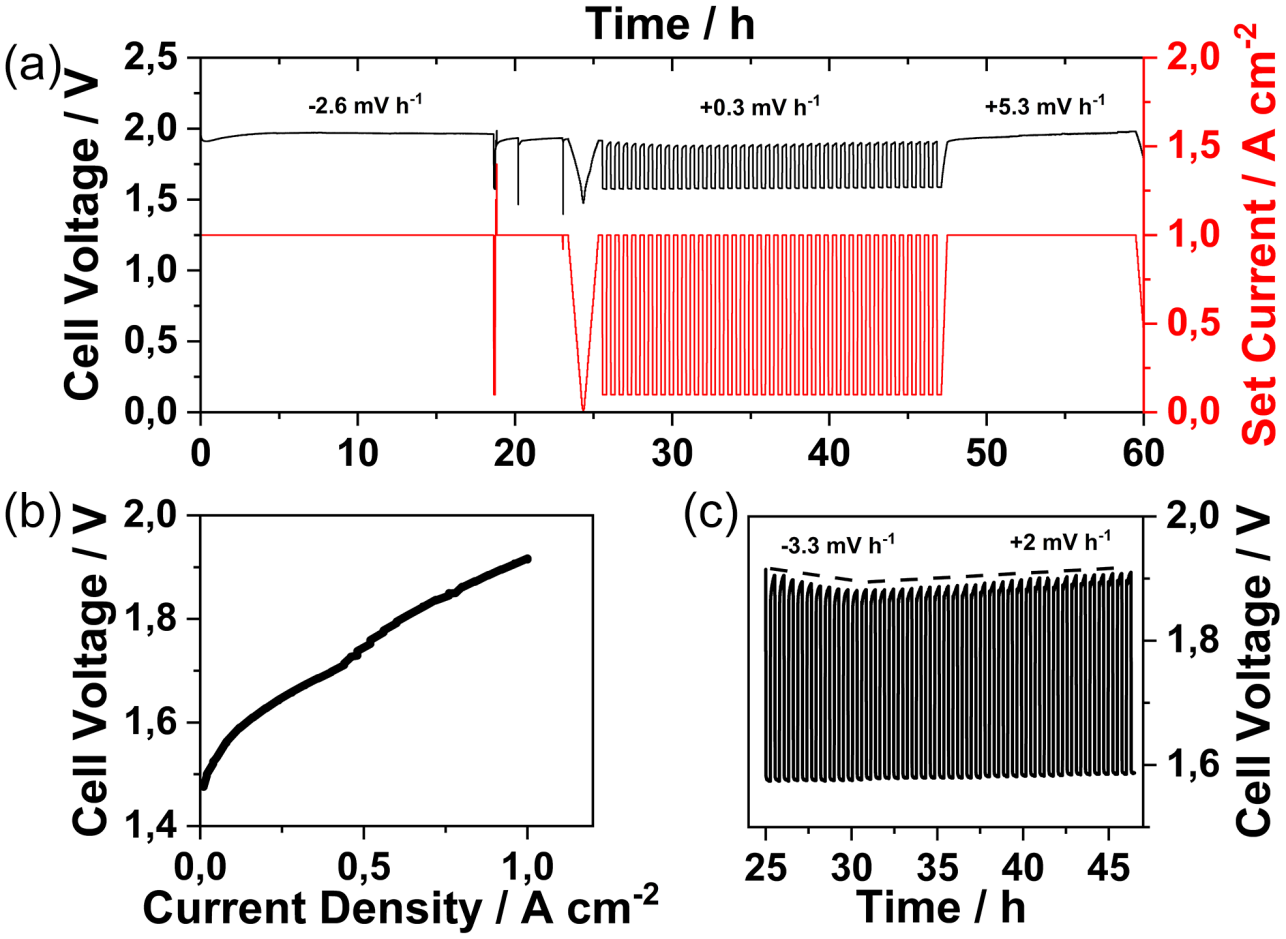
(a) AEMWE single‐cell test in 1.0M KOH at 60 °C over a total of 60 h under varied current densities with two stainless steel‐based electrodes: HER‐NiMo@ss, OER‐anodized Ni ED ss; (b) polarization curve recorded after 24 h of break‐in phase; and (c) inset of the accelerated stress test (AST) cycling between 1 A cm^−2^ and 0.1 A cm^−2^ for a total of 24 h.

To investigate the applicability of the synthesized electrodes in industrial electrolyzer setups, an AEMWE single‐cell measurement was carried out at 5x5 cm^2^ electrode size and with Ni flow fields (Figure S3). NiMo@ss HER electrode and anodized Ni@ss OER were assembled ionomer‐free into the cell. A AF3‐HWC9‐70‐0A4‐0005‐X Aemion+ reinforced anion exchange membrane (Ionomr) was used as the anion conducting membrane. The performance and stability under industrially relevant conditions of the catalysts were tested in a 1.0 M KOH asymmetric electrolyte feed and are shown in Figure 5.

The 60 h AEMWE‐single cell stability measurement depicted in Figure 5 gives insight into the electrochemical performance under dynamic and steady‐state operating conditions. It can be split up into four phases (1) break‐in phase or activation phase for the first 24 h, (2) polarization curve measurement after 24 h of activation, (3) AST cycling the current density between 1 and 0.1 A cm^−2^ for a total of 24 h, and (4) constant current holding phase at 1 A cm^−2^. The first 24 h of electrolyzer operation are represented by an activation phase in which the cell voltage decreases by −2.6 mV h^−1^. This activation occurs because of a reduction of interfacial contact resistance and establishment of ionic pathways.

After the initial break‐in period, a polarization curve was recorded reaching a cell voltage of 1.92 V at 1 A cm^−2^ at 60 °C in 1.0 M KOH, showing excellent performance with steel based PGM‐free materials (Figure 5b). This is comparable to other state‐of‐the art systems [11, 22, 36].

To simulate realistic operation scenarios associated with renewable energy sources such as solar and wind power, an AST was performed. Over a 24 h period, the current density was cycled between 1 and 0.1 A cm^−2^. These changes in current density can cause severe degradation to the cell components, which need to be mitigated. Despite these harsh conditions, the system only showed a minor total degradation rate of +0.3 mV h^−1^ (Figure 5a,c). The AST in Figure 5c shows two distinct regions or slopes. The first region from hour 25–31 is characterized by a −3.3 mV h^−1^ decrease in cell voltage. This shows that even after the 24 h activation and polarization curve protocol, the system wasn’t yet fully activated. In the second phase, an increase in cell voltage of +2.0 mV h^−1^ can be observed, indicating the end of the activation and the beginning of the degradation phase. This excellent stability under fluctuating load conditions shows the applicability to renewable energy systems and proves the strength in the electrode design.

In phase 4, a constant current hold at 1 A cm^−2^ was carried out that showed a significantly higher degradation of +5.3 mV h^−1^, which is in contrast to the stability during dynamic current cycling. This accelerated degradation under constant high current density is most likely associated with progressive membrane dehydration on the cathode side. Insufficient humidification can lead to increased ionic resistance and ultimately lead to membrane failure, which is confirmed by the holes in the membrane in Figure S4, Supporting Information.

The data indicates that, while the system tolerates dynamic operation well, prolonged steady‐state operation at high current densities requires careful water management. In particular, enhanced cathode humidification is necessary for long‐term experiments to prevent membrane drying and associated performance losses.

## 4 Summary and Conclusion

We have demonstrated a fully PGM‐free, steel‐based electrode concept for alkaline water electrolysis that combines earth‐abundant materials with scalable processing strategies. By integrating Ni ED with hydrothermal NiMo growth and reductive annealing, a hierarchical NiMo@ss architecture was obtained, consisting of MoO_2_‐derived needle‐like structures decorated with metallic Ni species and well‐defined NiMo interfaces, as confirmed by SEM–EDX, FIB–SEM, XRD, and XPS analyses. The engineered catalyst exhibited significantly enhanced hydrogen evolution activity in 0.1 M KOH, reaching an overpotential of −70 mV at −10 mA cm^−2^ and a Tafel slope of 132 mV dec^−1^. The improved kinetics are attributed to the synergistic interaction between Ni and Mo, promoting water dissociation in alkaline media, together with the hierarchical morphology that maximizes accessible active sites and facilitates mass transport. In addition, to boost the activity of the PTL for OER, a simple and efficient Ni ED and anodization procedure was carried out. The electrode shows an overpotential of 284 mV at 10 mA cm^−2^ in 0.1 M KOH.

Importantly, the electrodes were successfully implemented in a fully PGM‐free, ionomer‐free AEMWE single cell with an electrode area of 5 × 5 cm^2^, filling the gap between lab scale to industrially relevant dimensions, and achieving 1 A cm^−2^ at 1.92 V in 1 M KOH at 60 °C. The system showed great stability under dynamic current operation, only being limited by the lack of humidification on the cathode side leading to severe degradation at high constant current. Future studies will focus on improving cycling stability and activity further by: (1) incorporating an ionomer interlayer on the electrode to improve ionic conductivity and contact to the membrane (2) humidifying the cathode compartment to decrease the chance of the membrane drying out [37].

The combination of earth‐abundant elements, simplified electrode design, and competitive single‐cell performance highlights the potential of steel‐supported systems as a resource‐efficient and economically viable pathway toward large‐scale, sustainable hydrogen production.

## Author Contributions

**Lukas Heinius:** conceptualization, methodology, investigation, writing – original draft, writing – review & editing, formal analysis. **Pierre Schröer:** conceptualization, investigation, writing – original draft, methodology, writing – review & editing, formal analysis. **Vincent Wilke:** investigation, validation, formal analysis, writing – review & editing. **Merel Rittel:** writing – review & editing, investigation, formal analysis. **Quy Duy Doan:** investigation, writing – review & editing, formal analysis. **Chen Yie Thum:** investigation, formal analysis, writing – review & editing. **Raffaele Amitrano:** investigation, formal analysis, writing – review & editing. **Christian M. Günther:** investigation, formal analysis, writing – review & editing. **Johannes Schmidt:** writing – review & editing, investigation, formal analysis. **Duc Van Dinh:** writing – review & editing, investigation, formal analysis. **Dominik Dworschak:** writing – review & editing, investigation. **Aldo Saul Gago:** writing – review & editing. **Kaspar Andreas Friedrich:** writing – review & editing. **Fabio Dionigi:** writing – review & editing, conceptualization, supervision. **Peter Strasser:** writing – review & editing, conceptualization, supervision, funding acquisition, project administration.

## Funding

This study was supported by Bundesministerium für Wirtschaft und Energie (03SF0613B), Bundesministerium für Forschung, Technik und Raumfahrt (BMFTR, former BMBF) (03HY130A).

## Conflicts of Interest

The authors declare no conflicts of interest.

## Supporting information

Supplementary Material

## Data Availability

The data that support the findings of this study are available from the corresponding author upon reasonable request.
