# Supplementary material for: Steel Based Precious Group Metal‐Free High‐Performance Electrodes for Alkaline Exchange Membrane Water Electrolysis
Source: ChemSusChem. 2026 Jul 16;19(14):e70895. doi: 10.1002/cssc.70895 (PMC13376225; doi:10.1002/cssc.70895)
Supplement: Supplementary file 1 — Supplementary Material [file CSSC-19-e70895-s001.pdf]

Supporting information

Steel based precious group metal-free high-performance electrodes for Alkaline Exchange Membrane water electrolysis

Lukas Heinius,[a] Pierre Schröer,[a] Vincent Wilke,[b] Merel Rittel,[a] Quy Duy Doan,[a] Chen Yie Thum,[a] Raffaele Amitrano,[a] Christian M. Günther,[c] Johannes Schmidt,[d] Duc Van Dinh,[e] Dominik Dworschak,[f] Aldo Saul Gago,[b] Kaspar Andreas Friedrich,[b] Fabio Dionigi,[a*] Peter Strasser[a*]

[a]*Technische Universität Berlin, The Electrochemical Energy, Catalysis, and Materials Science Laboratory, Department of Chemistry, Straße des 17. Juni 135, 10623 Berlin, Germany*

[b]*Institute of Engineering Thermodynamics, German Aerospace Center (DLR), Stuttgart, Germany*

[c]*Technische Universität Berlin, Center for Electron Microscopy (ZELMI), Straße des 17. Juni 135, 10623 Berlin, Germany*

[d]*Technische Universität Berlin, Department of Chemistry, Functional Materials, Straße des 17. Juni 135, 10623 Berlin, Germany*

[e]*Paul-Drude Institute for Solid-State Electronics (PDI), Hausvogteiplatz 5-7, 10117 Berlin*

[f]*Helmholtz Institute Erlangen-Nürnberg for Renewable Energy (IET-2), Forschungszentrum Jülich, Cauerstr. 1, 91058 Erlangen, Germany*

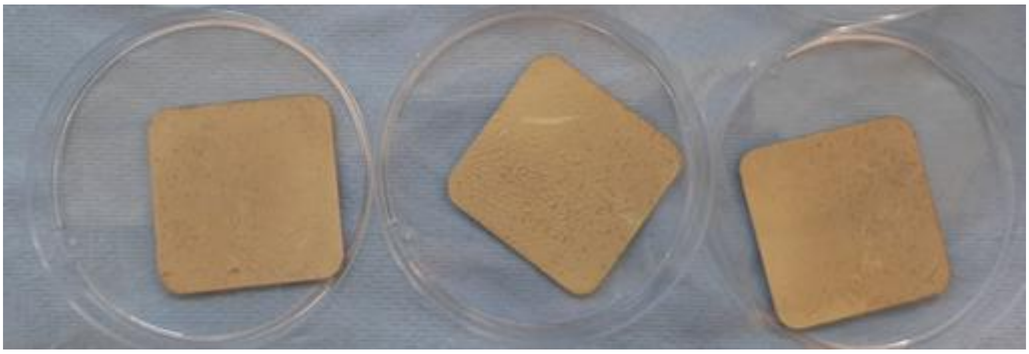

Figure 1: 25cm^2 NiMo on stainless steel after hydrothermal deposition and before the final thermal reduction step.

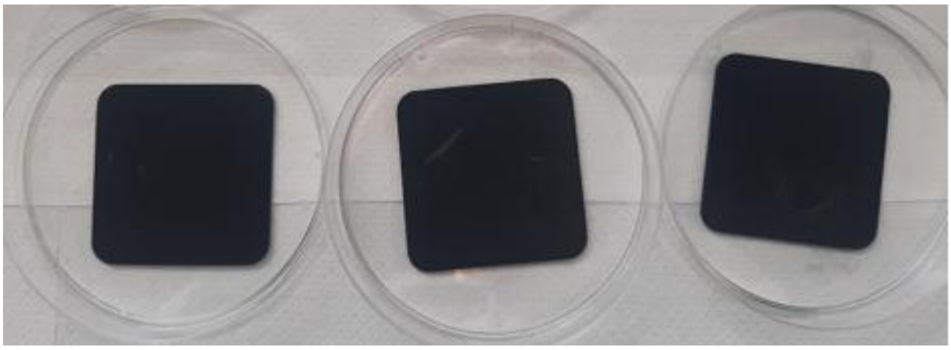

Figure 2: 25cm^2 NiMo on stainless steel (NiMo@ss) HER catalyst after H2 reduction.

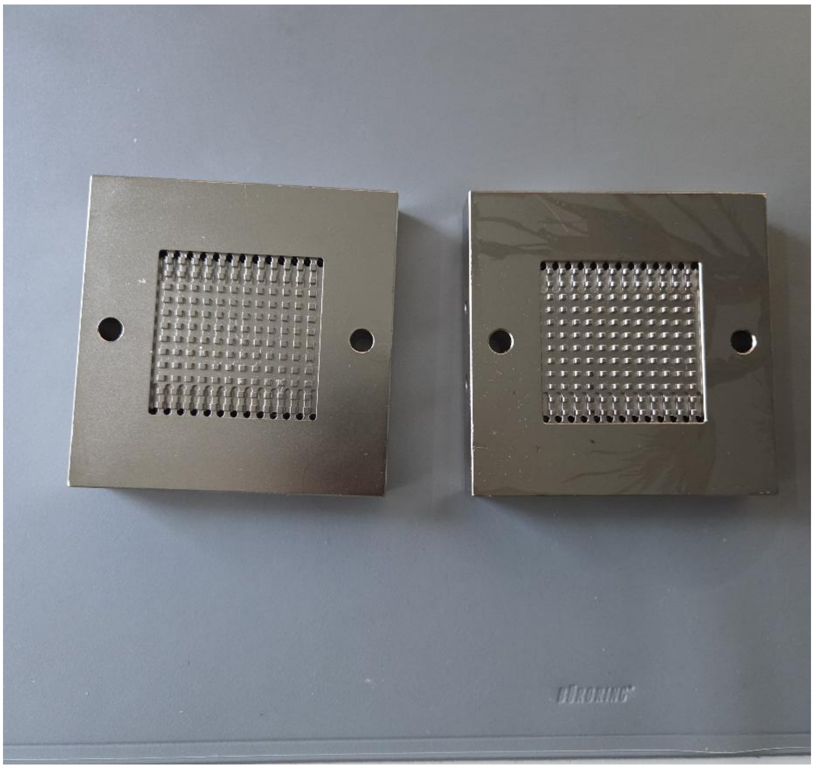

Figure 3: 25cm^2 Nickel flow fields used for the AEMWE single-cell measurements.

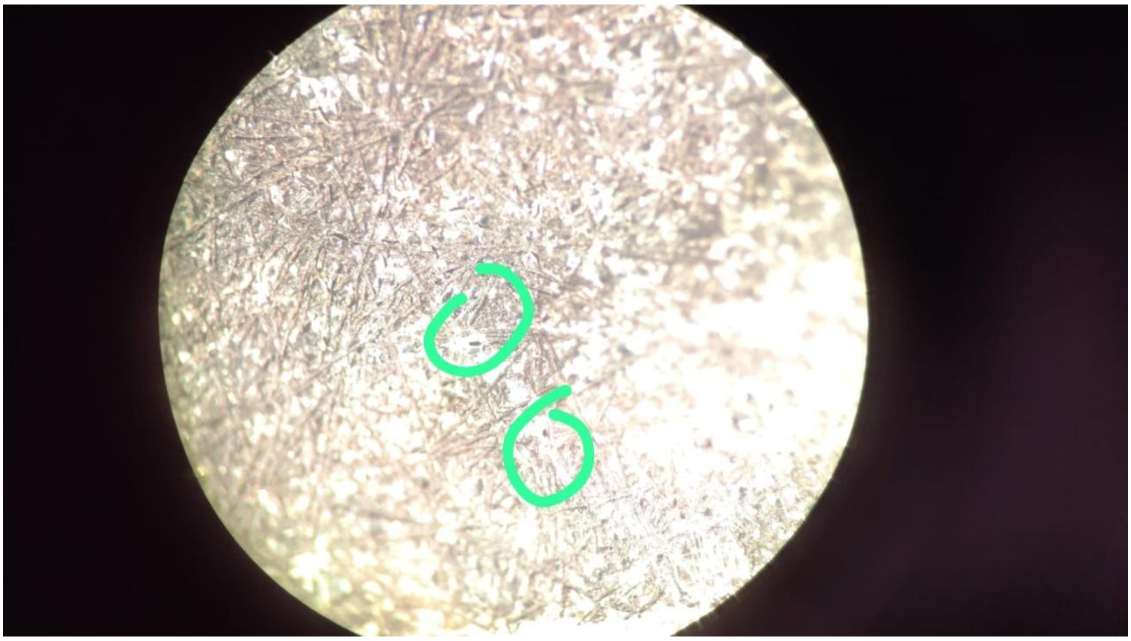

Figure 4: Light microscope images of membrane after cell testing.

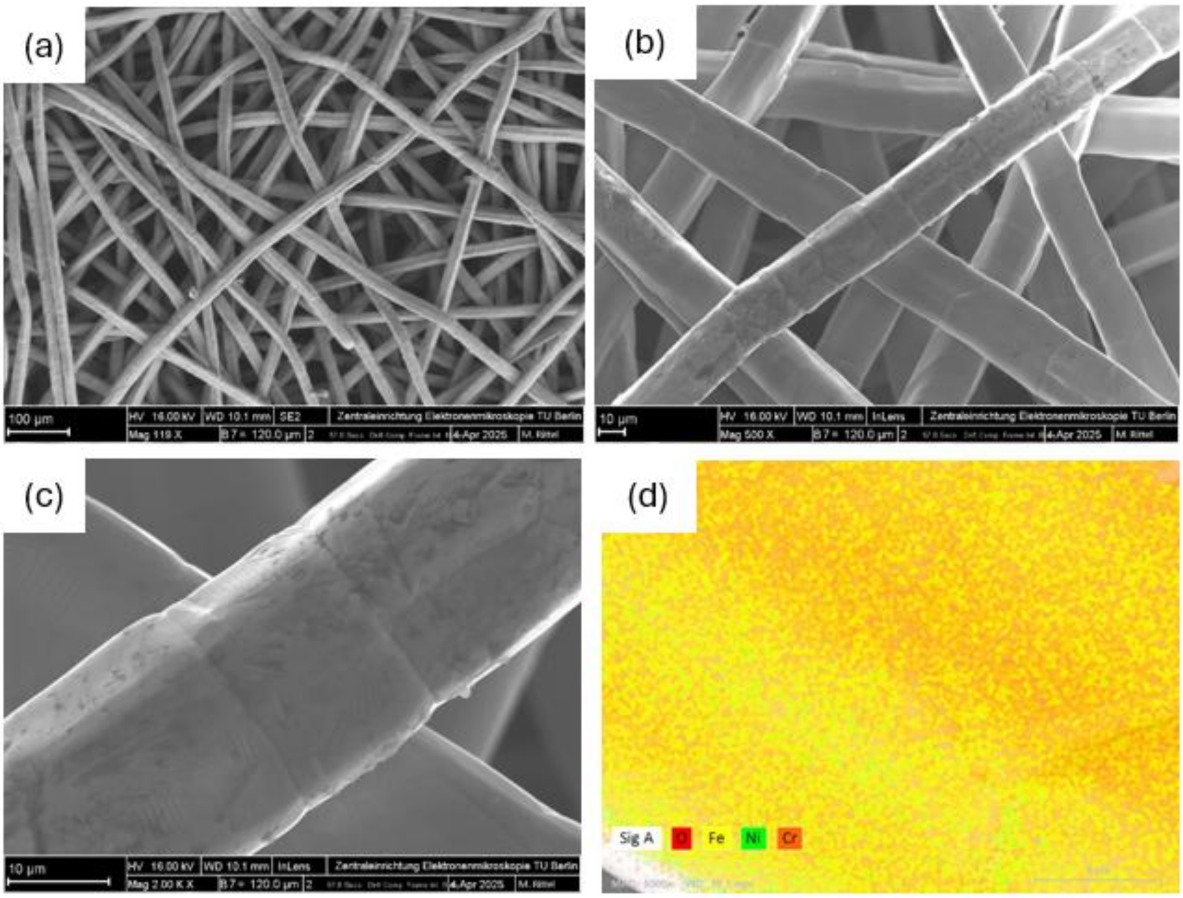

Figure 5: SEM and SEM EDX images of the raw stainless-steel substrate at different magnifications (a-c) and EDX mapping (d).

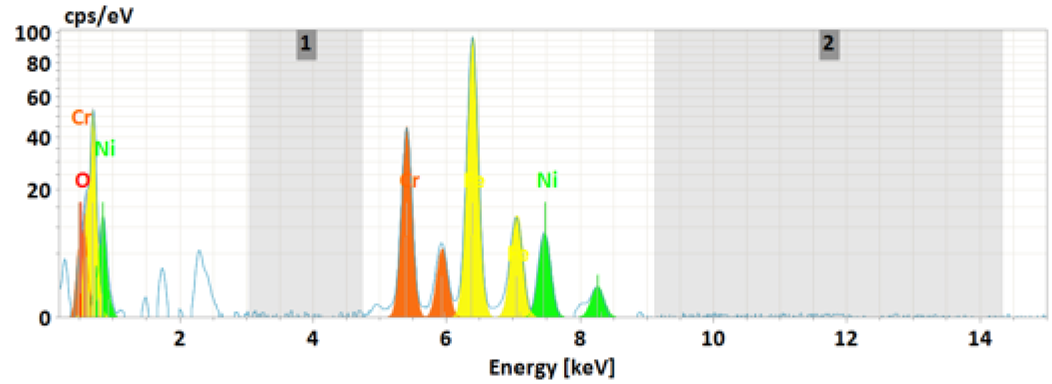

Map.spx

Element	At. No.	Line s.	Netto	Mass Norm. [%]	Atom [%]
O	8	K-Serie	0	0,00	0,00
Fe	26	K-Serie	6764620	71,27	70,66
Ni	28	K-Serie	640911	10,33	9,75
Cr	24	K-Serie	2908263	18,40	19,60
			**Sum**	**100,00**	**100,00**

Figure 6: EDX mapping results of the SEM images of raw stainless steel.

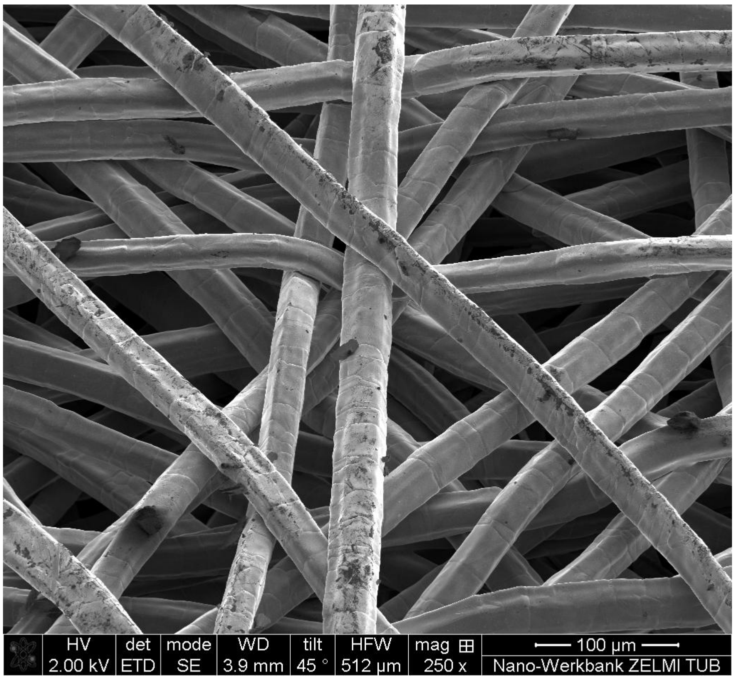

Figure 7: SEM image of raw stainless-steel at a magnification of 250x.

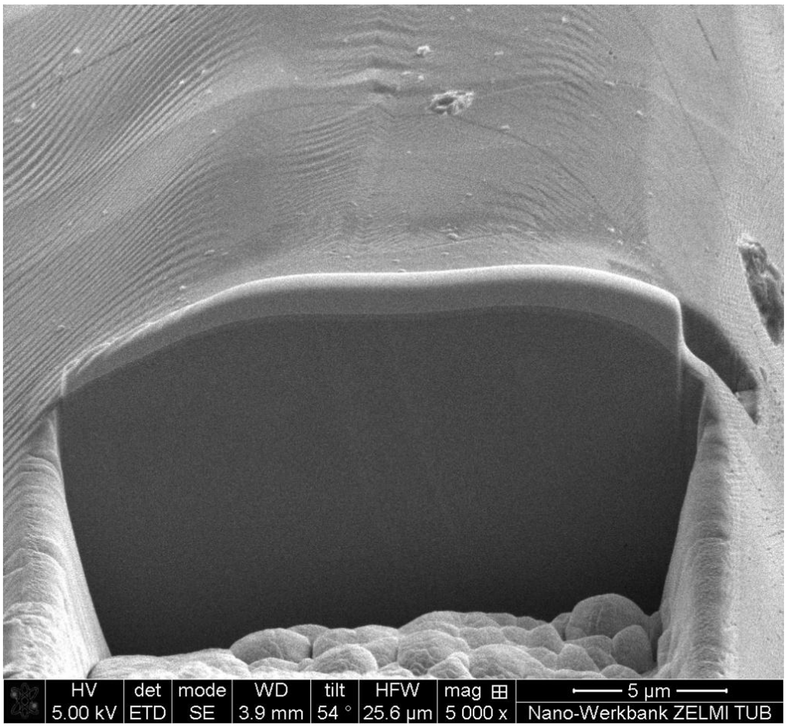

Figure 8: FIB SEM cut of untreated stainless steel at a magnification of 5000x.

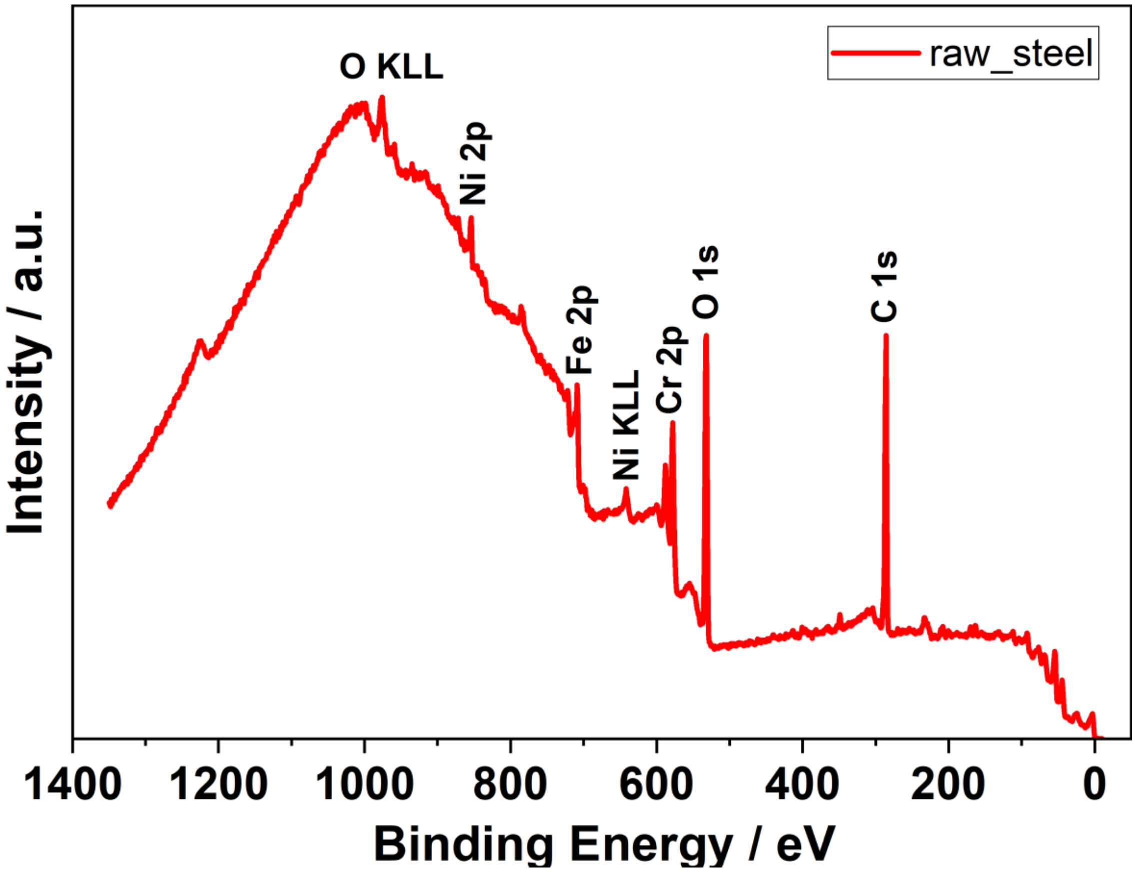

Figure 9: XPS survey scan of the untreated stainless steel.

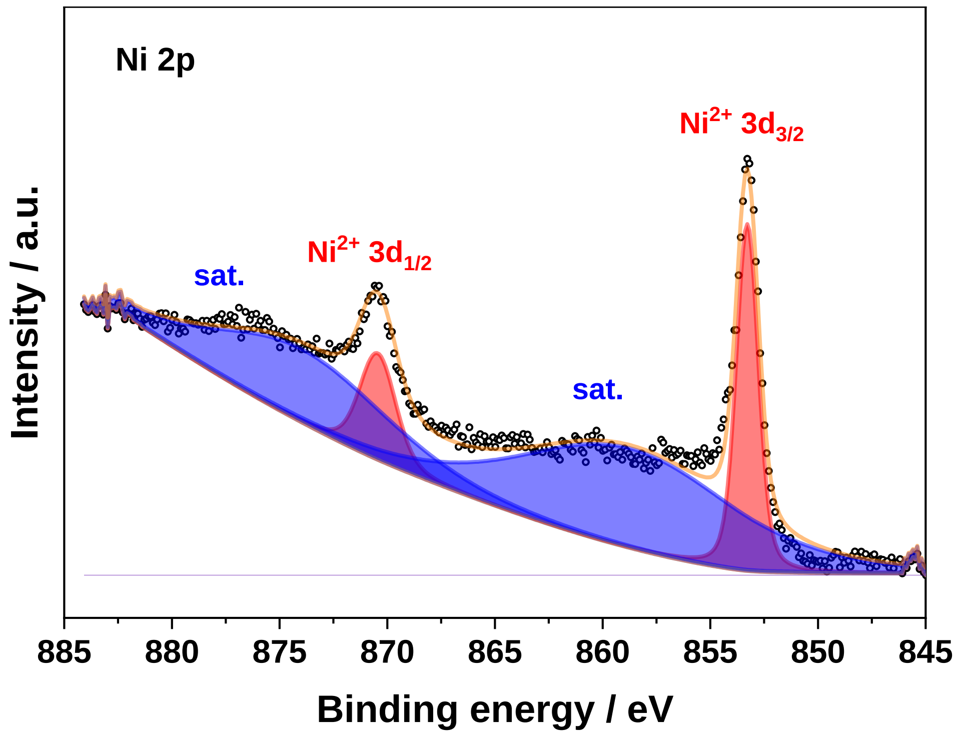

Figure 10: High-resolution XPS Ni 2p spectra with component fit of raw stainless steel.

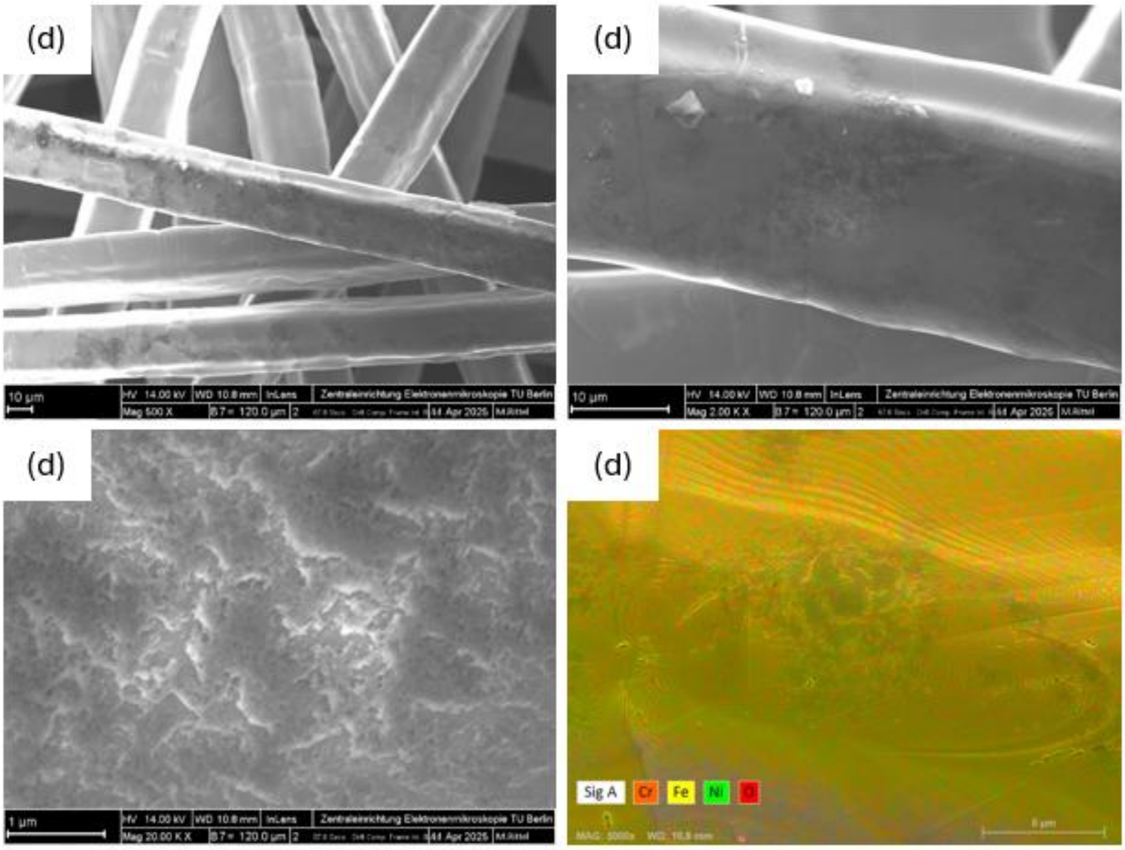

Figure 11: SEM images of the stainless PTLs after anodization process at different magnifications a) b) c) and EDX mapping d).

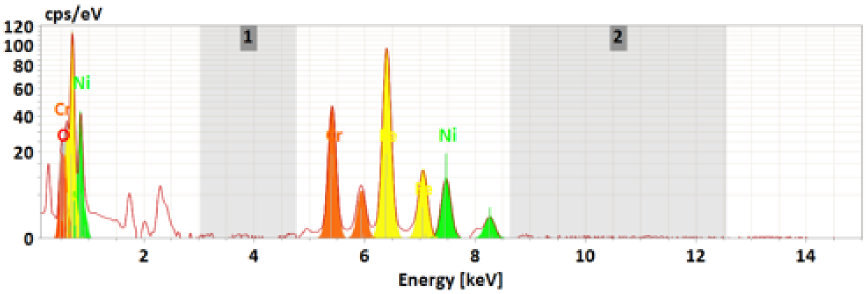

Map.txt

Element	At. No.	Line s.	Netto	Mass Norm. [%]	Atom [%]
O	8	K-Serie	424538	1,67	5,55
Cr	24	K-Serie	3345528	17,64	18,06
Fe	26	K-Serie	7430867	68,67	65,48
Ni	28	K-Serie	787481	12,02	10,91
			**Sum**	**100,00**	**100,00**

Figure 12: EDX Mapping of anodized stainless steel.

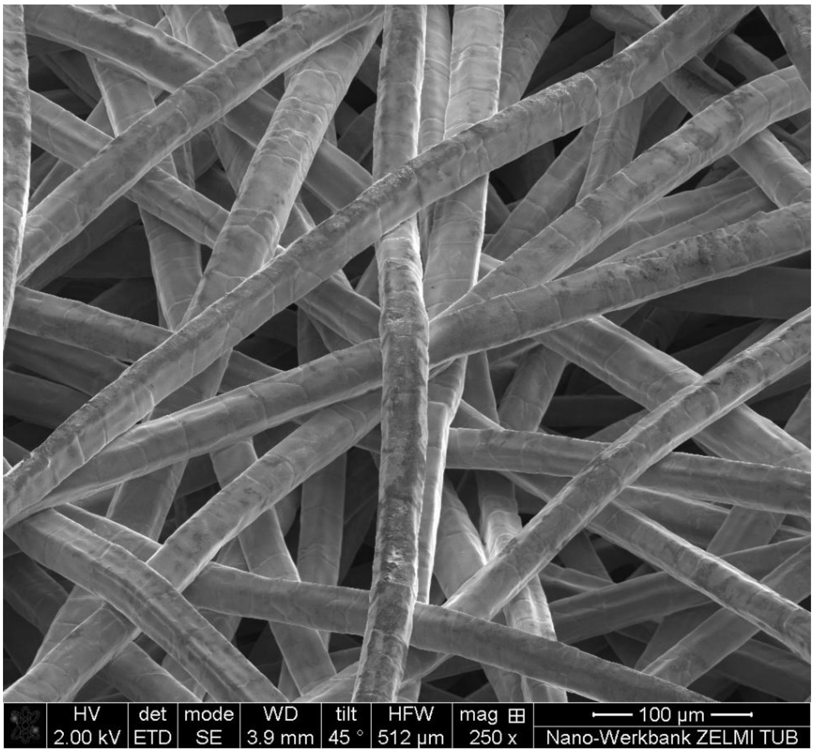

Figure 13: SEM image of anodized stainless steel at a magnification of 250x.

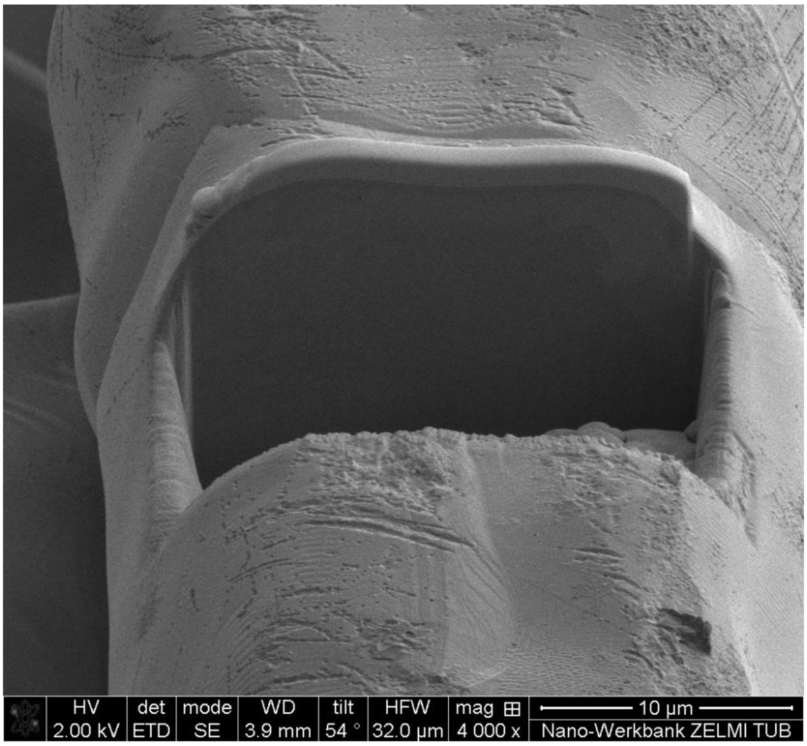

Figure 14: FIB-SEM image of anodized stainless steel at a magnification of 4000x.

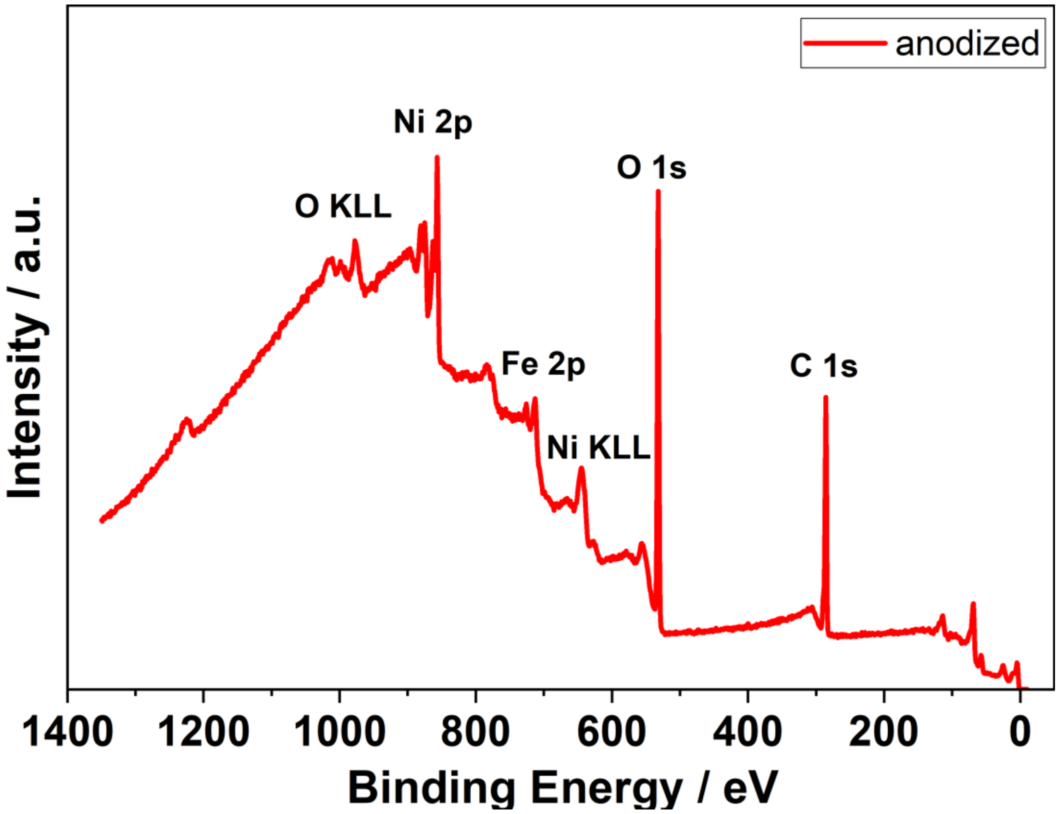

Figure 15: XPS survey scan of stainless steel after anodization.

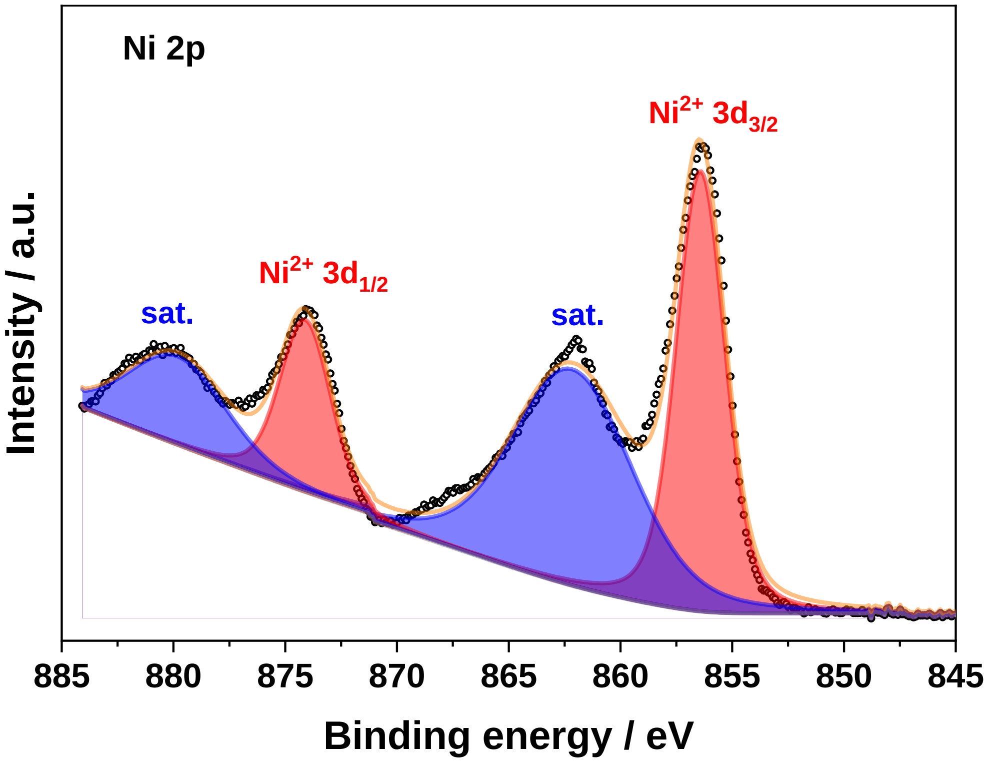

Figure 16: High-resolution XPS Ni 2p spectra with component fit of stainless steel after anodization.

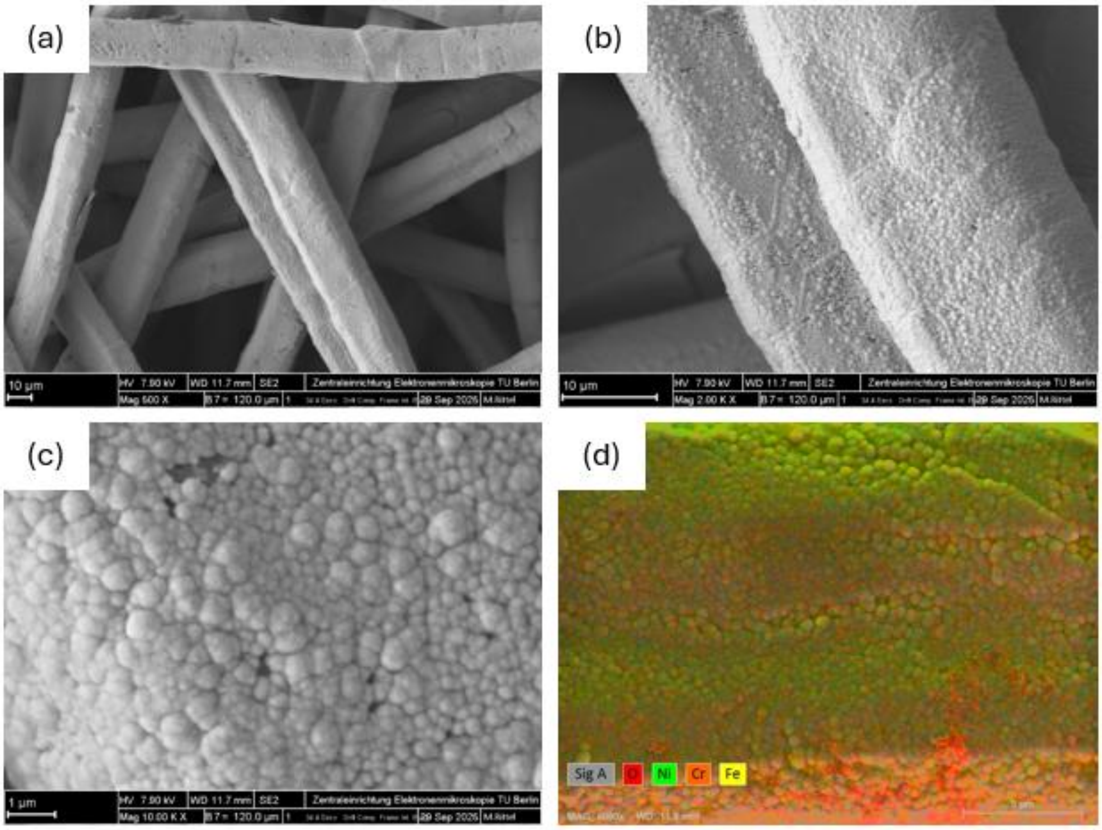

Figure 17: SEM images of the Electrodeposited steel at different magnifications a) b) c) and EDX mapping d).

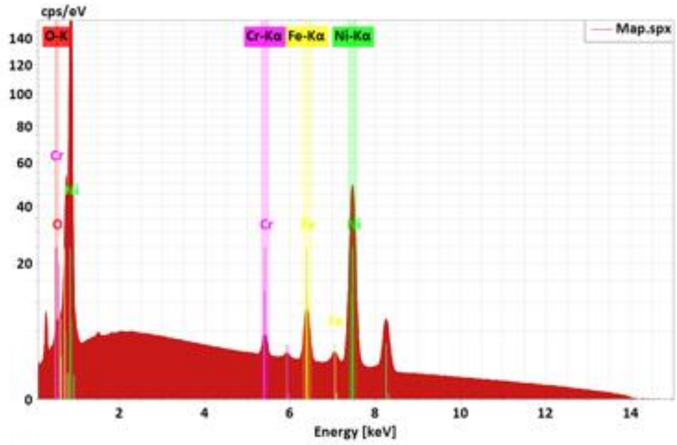

Map.spx

Element	At. No.	Line s.	Netto	Mass [%]	Mass Norm. [%]	Atom [%]
Fe	26	K-Serie	146522	6,20	6,70	7,01
Cr	24	K-Serie	44100	1,33	1,43	1,61
Ni	28	K-Serie	1004331	85,01	91,87	91,38
O	8	K-Serie	0	0,00	0,00	0,00
			**Sum**	**92,54**	**100,00**	**100,00**

Figure 18: EDX mapping results of electrodeposited steel.

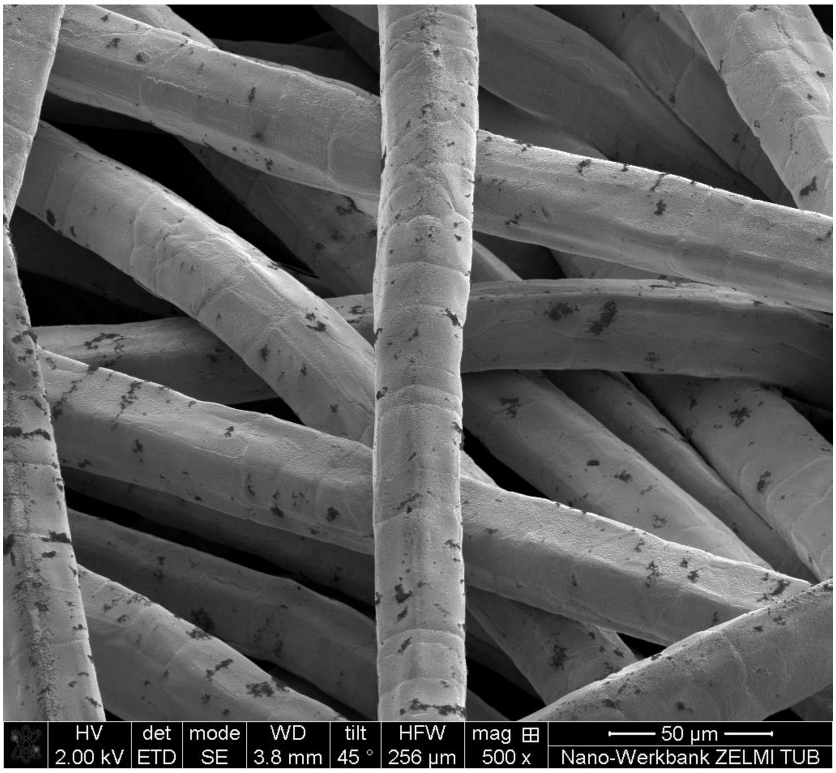

Figure 19: SEM of electrodeposited steel at magnification 500x.

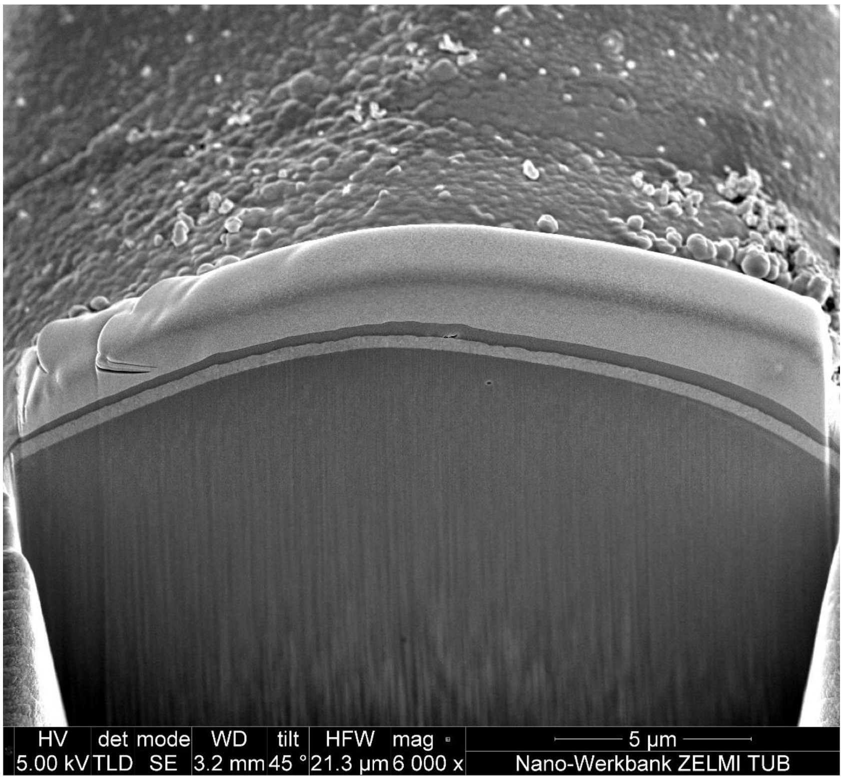

Figure 20: FIB-SEM of electrodeposited steel at magnification 6000x.

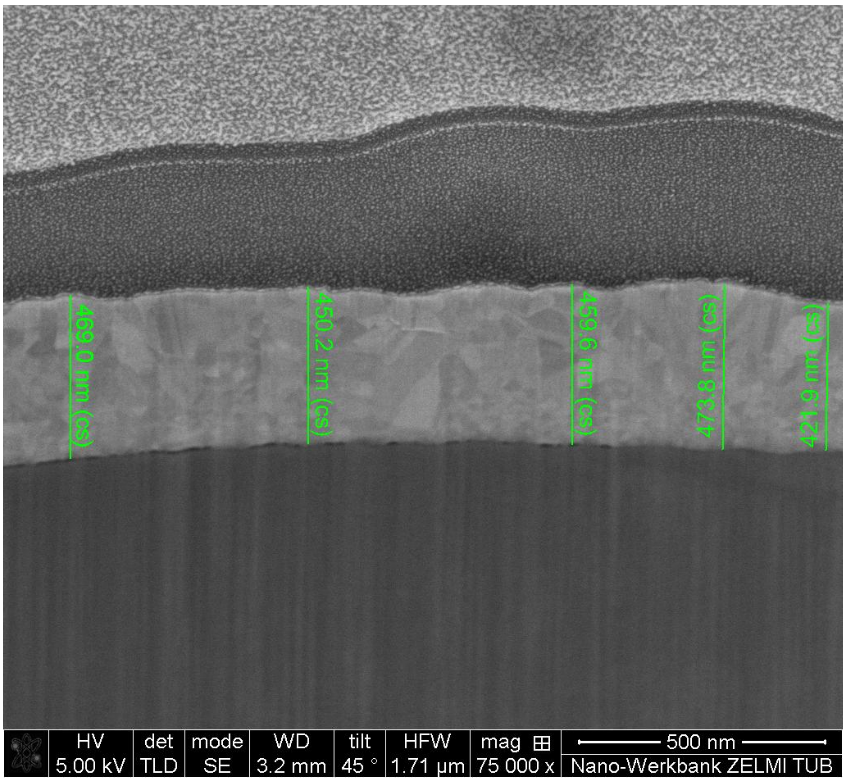

Figure 21: FIB-SEM electrodeposited steel at magnification 7500x with an average Ni layer thickness of 455 nm after 30s of electrodeposition on a 2cm^2 sample.

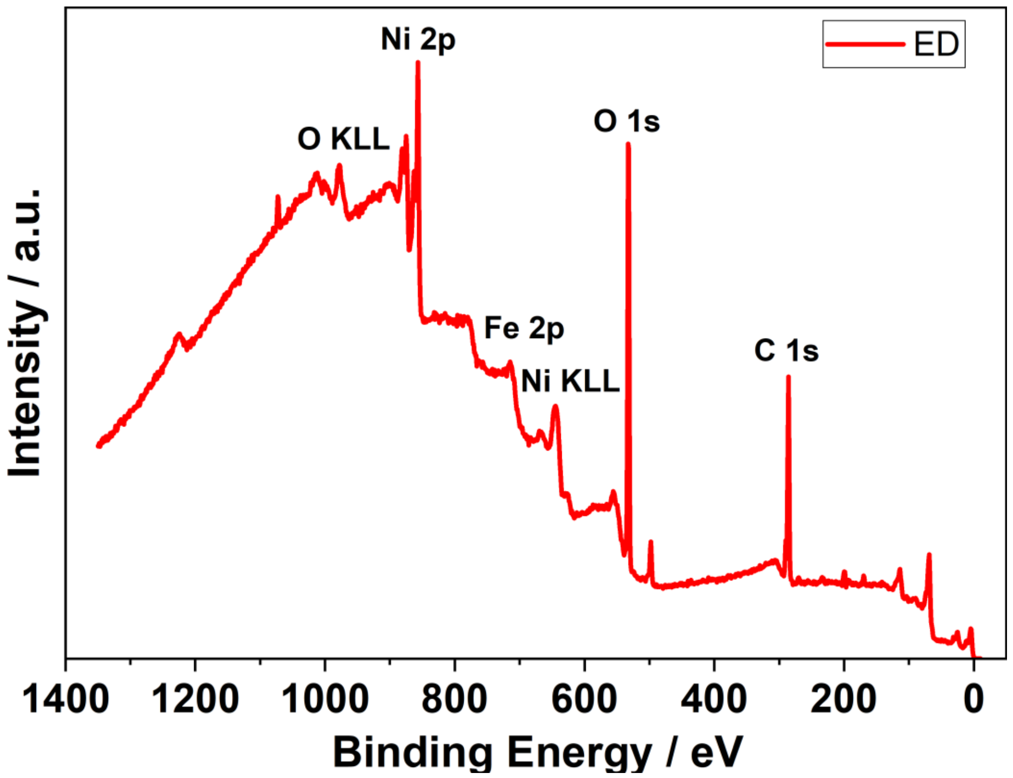

Figure 22: XPS survey scan of the Electrodeposited steel.

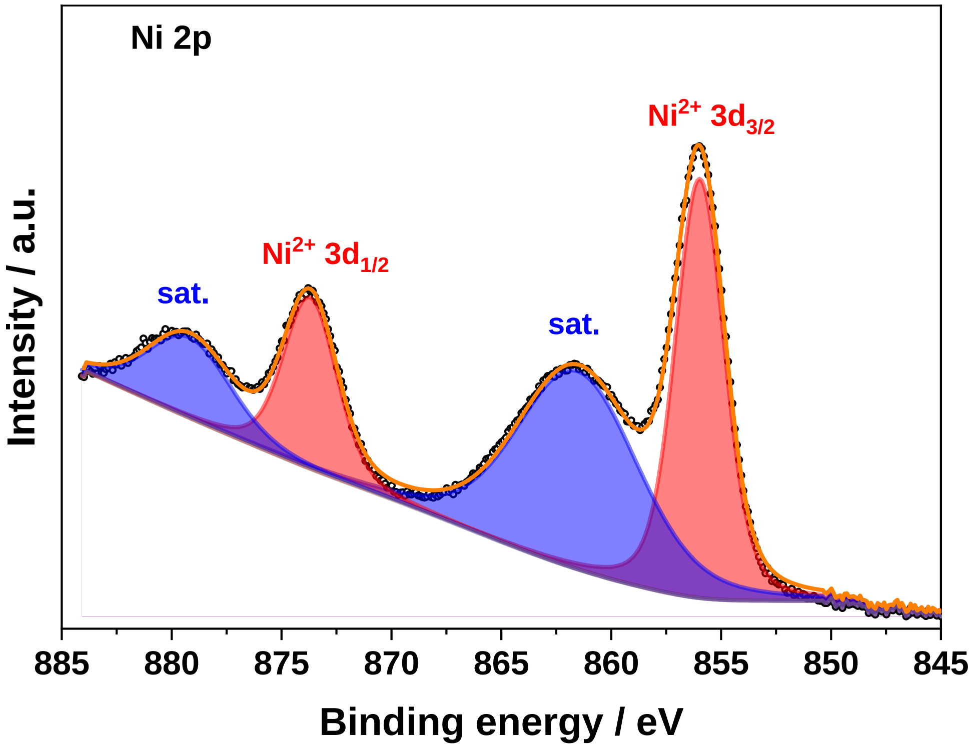

Figure 23: High-resolution XPS Ni 2p spectra with component fit of electrodeposited steel.

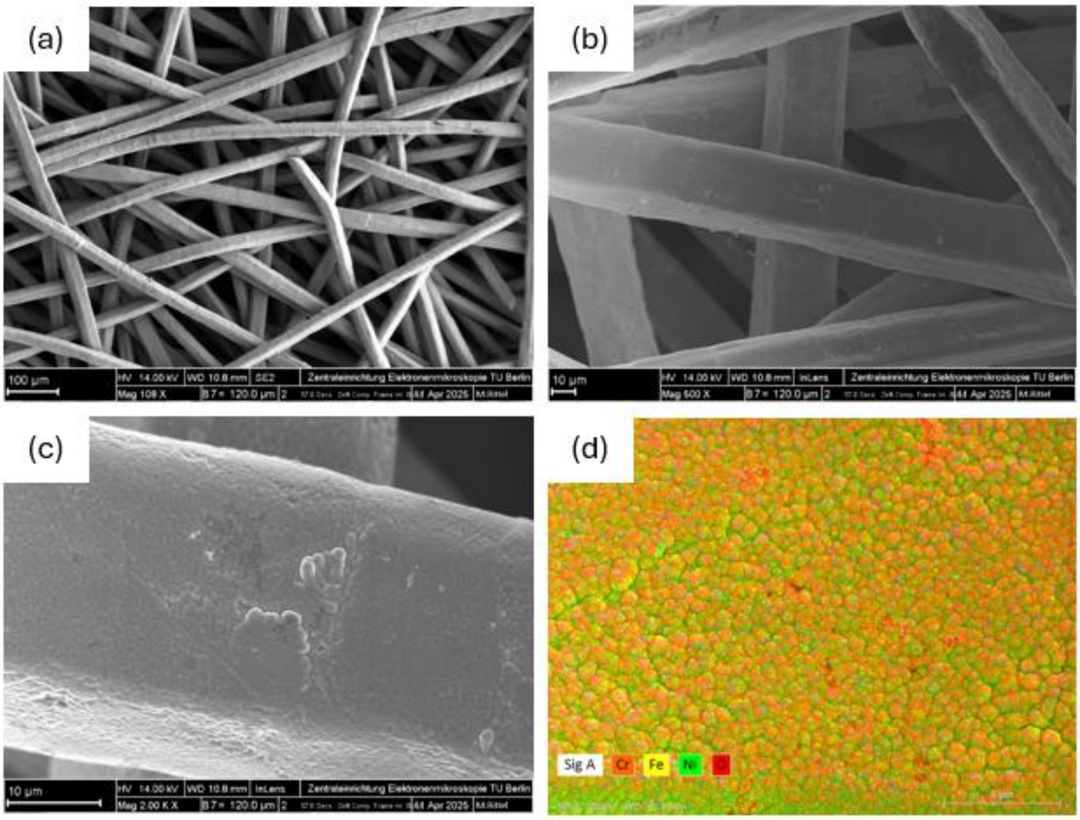

Figure 24:SEM images of the electrodeposited steel after the anodization process at different magnifications a) b) c) and EDX mapping d).

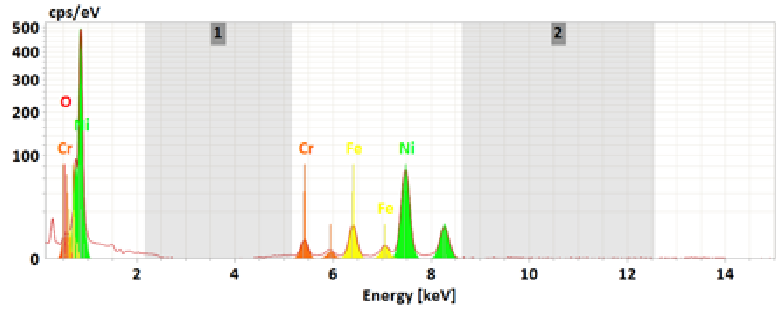

Map

Element	At. No.	Line s.	Netto	Mass Norm. [%]	Atom [%]
Cr	24	K-Serie	300650	1,24	1,37
Fe	26	K-Serie	1085608	6,22	6,43
Ni	28	K-Serie	8181052	92,11	90,64
O	8	K-Serie	125608	0,43	1,55
			**Sum**	**100,00**	**100,00**

Figure 25: EDX mapping of the electrodeposited steel after the anodization process.

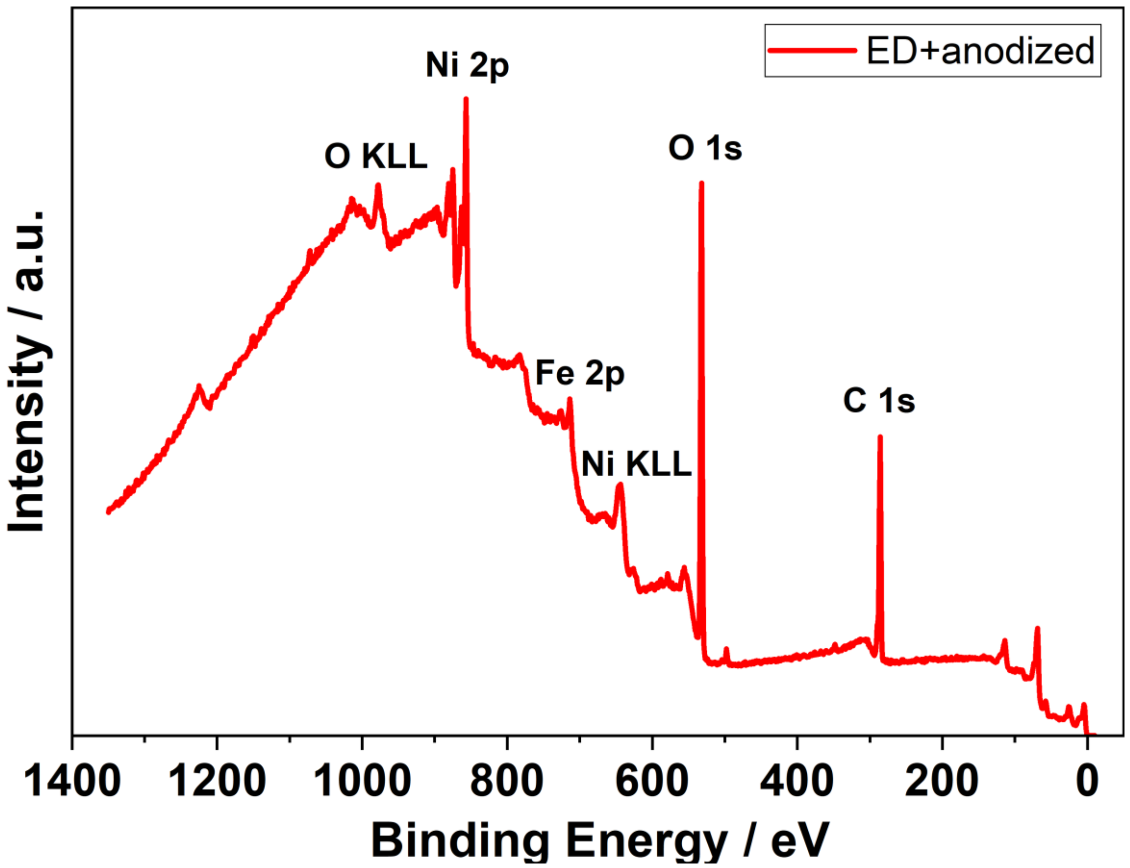

Figure 26: XPS survey scan of the electrodeposited steel after the anodization process.

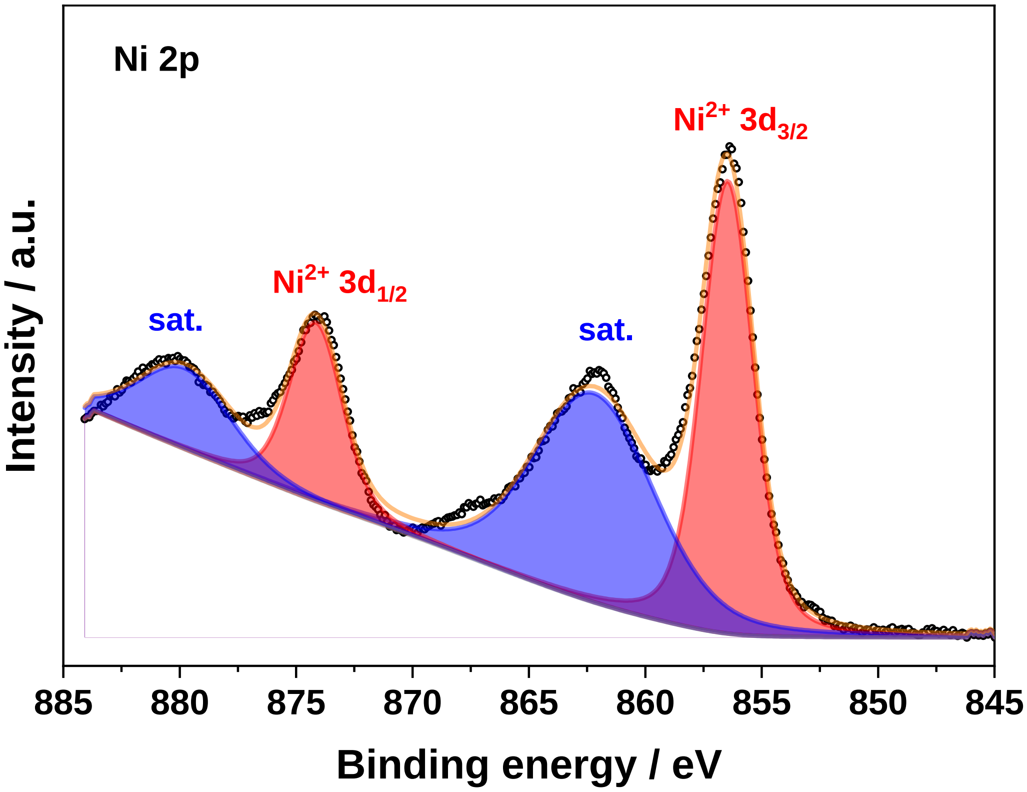

Figure 27: High-resolution XPS Ni 2p spectra with component fit of electrodeposited steel after the anodization process.

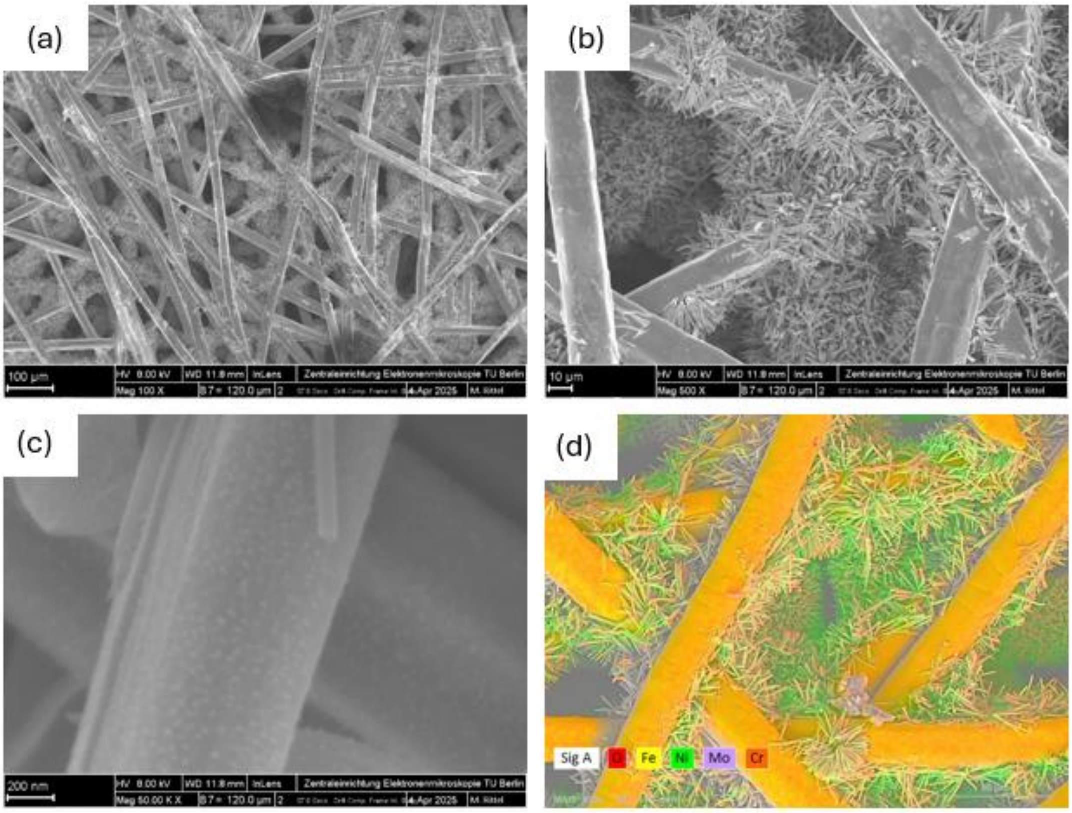

Figure 28: SEM image of the Nickel molybdenum deposited on the Nickel layered stainless steel magnifications a) b) c) and EDX mapping d).

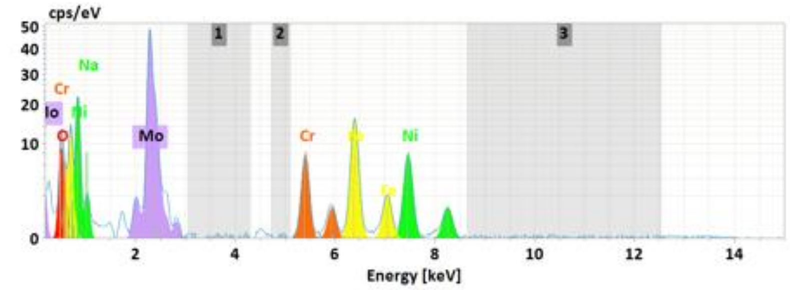

Map

Element	At. No.	Line s.	Netto	Mass Norm. [%]	Atom [%]
O	8	K-Serie	288272	6,03	20,66
Fe	26	K-Serie	1089264	27,23	26,73
Ni	28	K-Serie	564281	23,12	21,60
Mo	42	L-Serie	3181178	34,46	19,69
Cr	24	K-Serie	495490	7,90	8,33
Na	11	K-Serie	84728	1,26	3,00
			**Sum**	**100,00**	**100,00**

Figure 29: EDX mapping of the Nickel molybdenum deposited on the Nickel layered stainless steel.

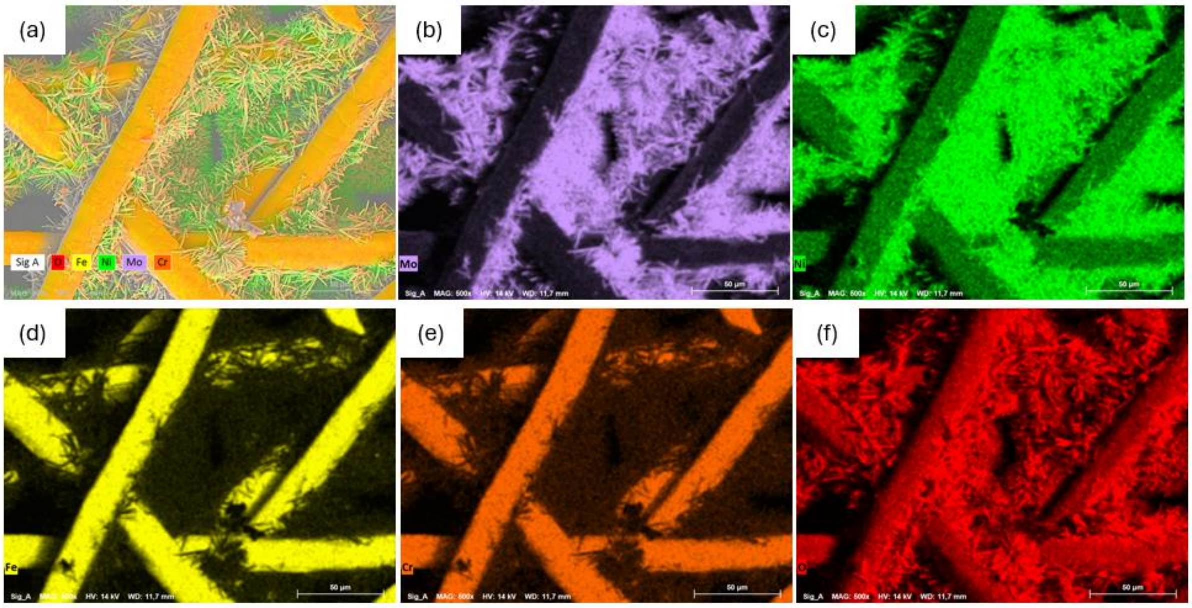

Figure 30: SEM image of Nickel molybdenum deposited on the Nickel layered stainless steel with EDX detector showing the different elements and their positions.

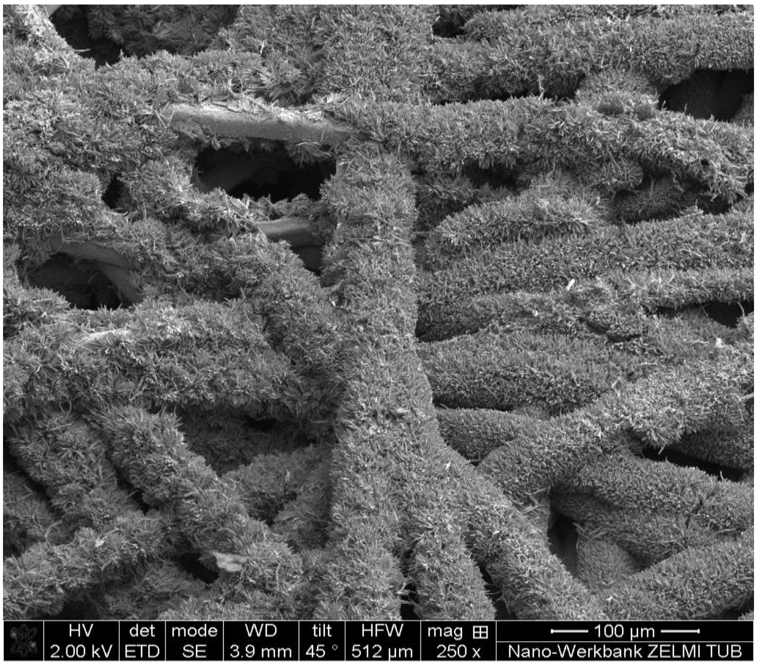

Figure 31: SEM image of the Nickel molybdenum deposited on the Nickel layered stainless steel with a magnification of 250x.

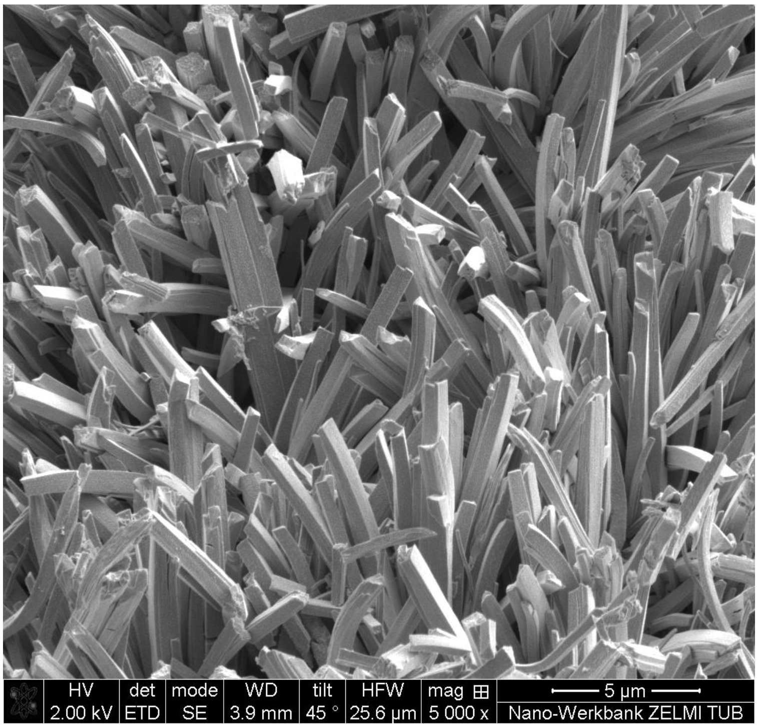

Figure 32: SEM at a magnification of 5000x image of the Nickel molybdenum deposited on the Nickel layered stainless steel, Magnification to the monolithic like structure of the MoO_2 phase.

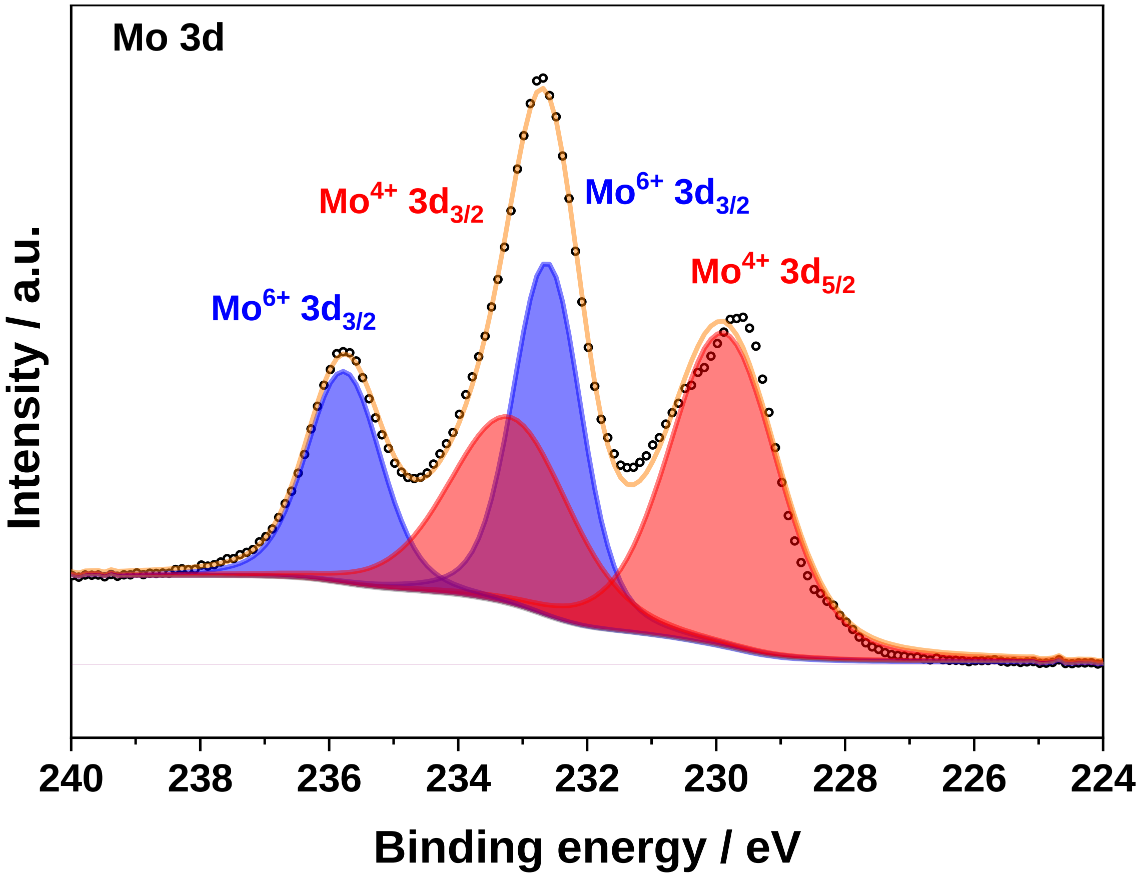

Figure 33: High-resolution XPS Mo 3d spectra with component fit of NiMo@ss (beginning of life – BEOL).

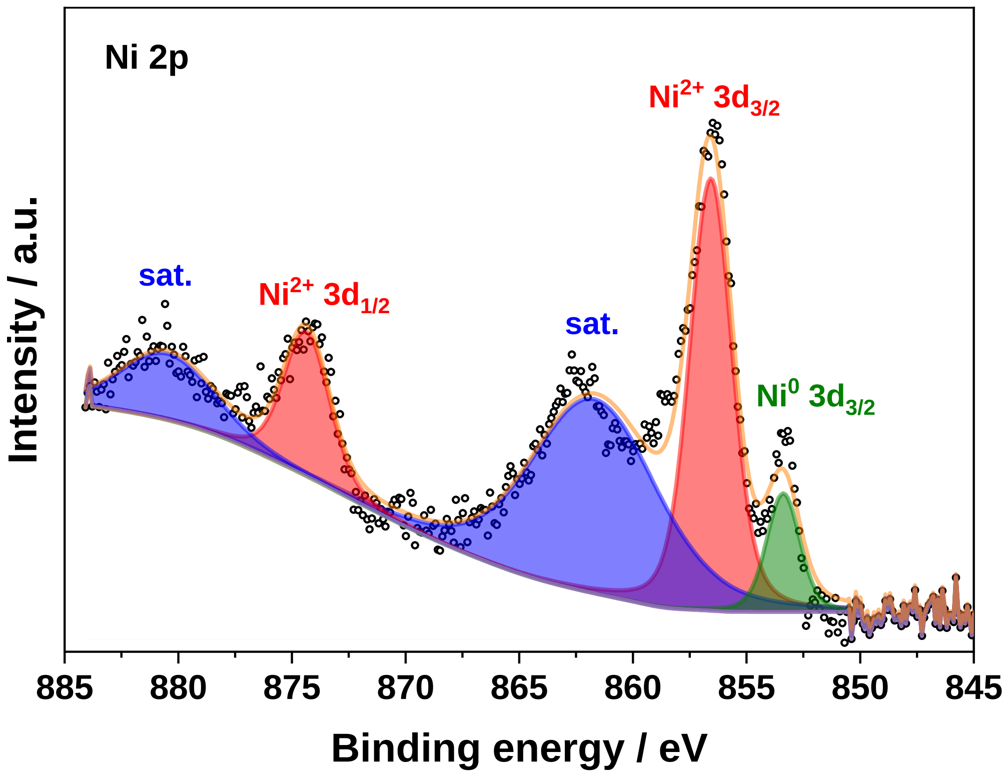

Figure 34: High-resolution XPS Ni 2p spectra with component fit of NiMo@ss (beginning of life – BEOL).

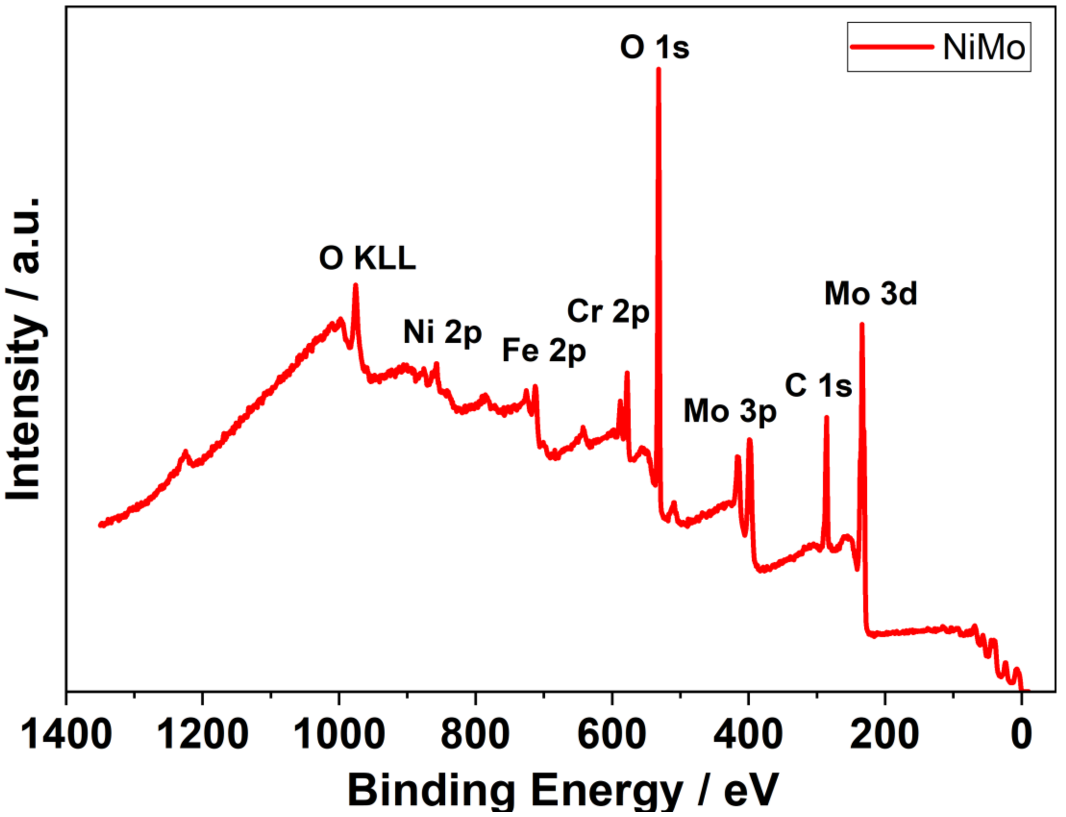

Figure 35: XPS survey scan of the Nickel molybdenum deposited on the Nickel layered stainless steel (beginning of life).

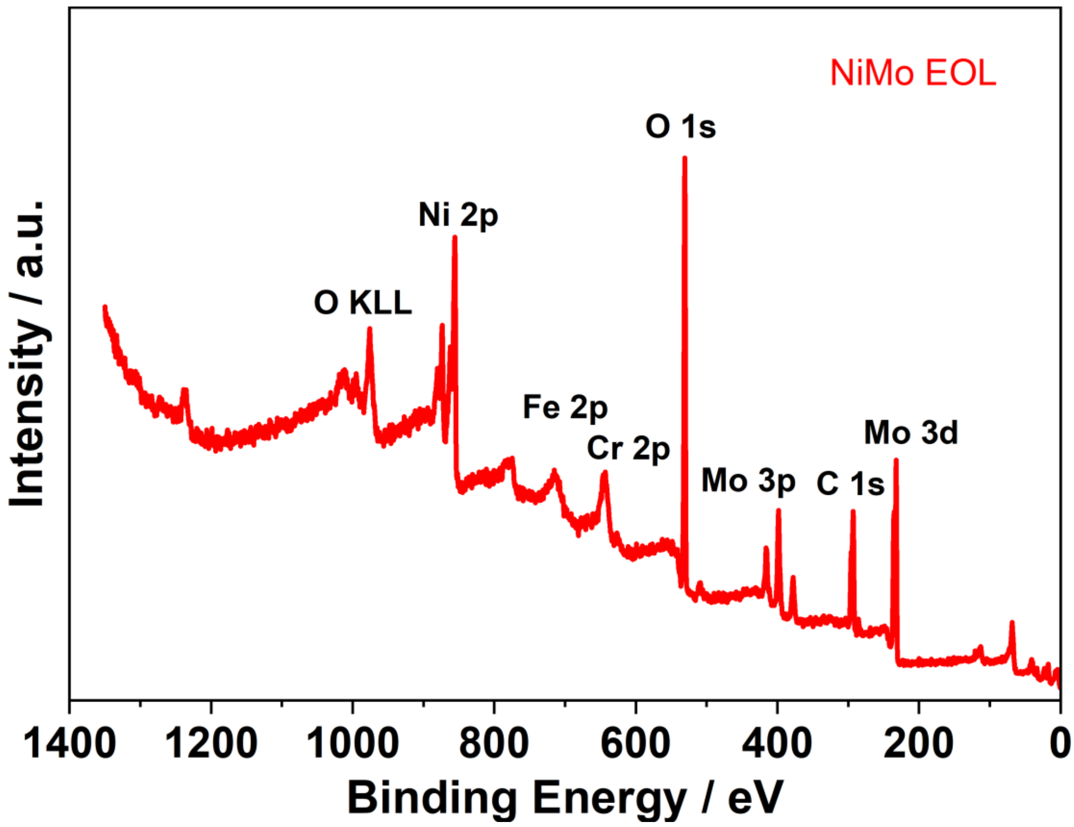

Figure 36: XPS survey scan of the Nickel molybdenum deposited on the Nickel layered stainless steel (end of life).

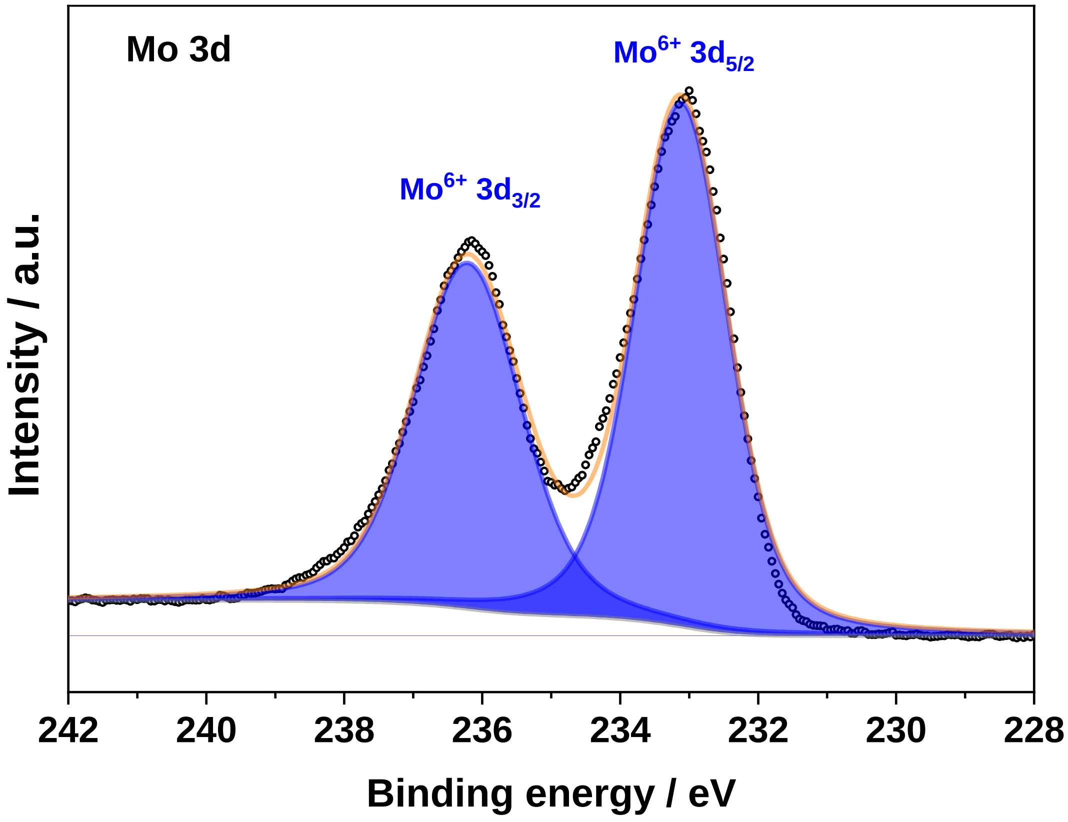

Figure 37: High-resolution XPS Mo 3d spectra with component fit of NiMo@ss (end of life – EOL).

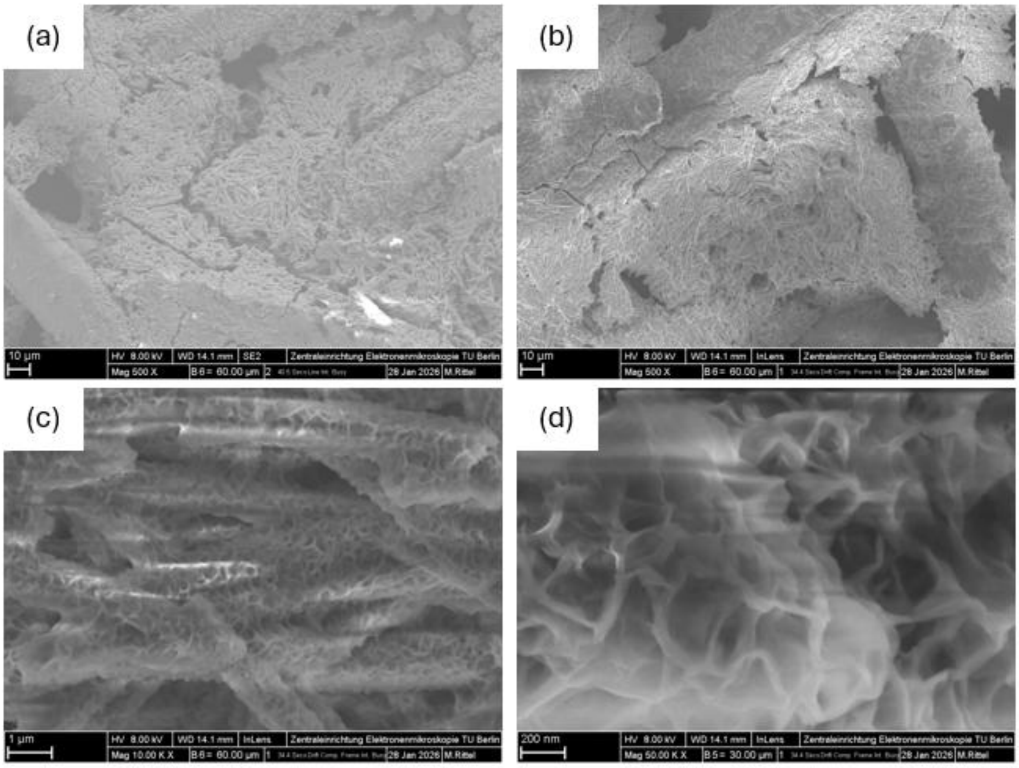

Figure 38: SEM images of the Nickel molybdenum deposited on the Nickel layered stainless steel after the AEMWE single-cell measurements (EOL) with magnification of a) 500x b) 500x, c) 10000x and d) 50000x.

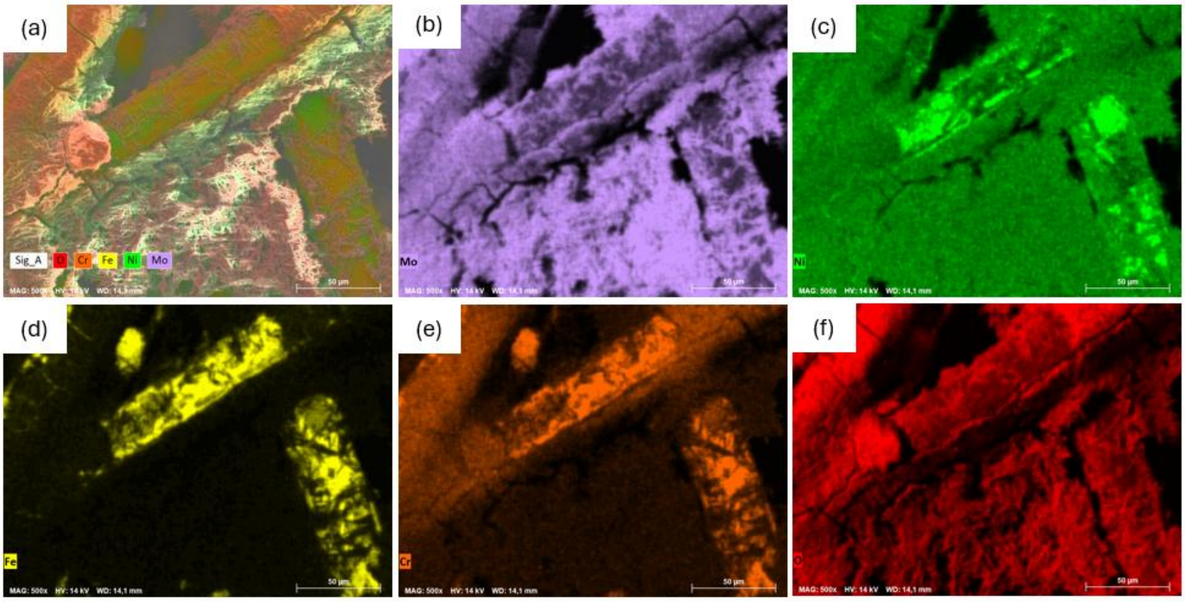

Figure 39: SEM image of Nickel molybdenum deposited on the Nickel layered stainless steel after the AEMWE single-cell measurements (EOL) with EDX detector showing the different elements and their positions.

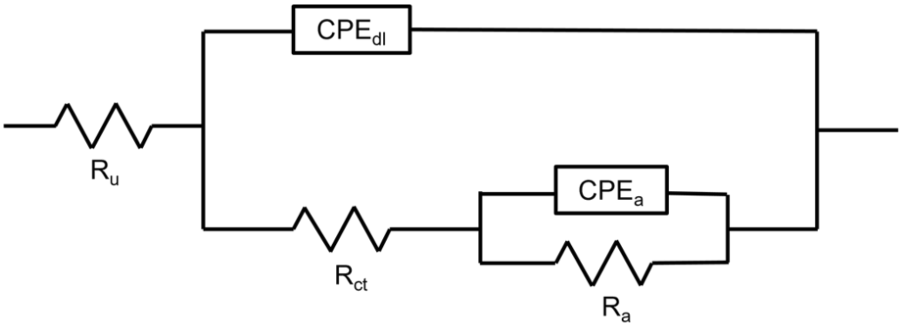

Figure 40: Schematic representation of the equivalent electric circuit for fitting the Nyquist plot obtained from electrochemical impedance spectroscopy (EIS) in this study. The equivalent electric circuit consists of uncompensated resistance (R_u), charge transfer resistance (R_{ct}), double layer capacitance described as constant phase element (CPE_{dl}), OER adsorbates resistance (R_a), and adsorbates capacitance described as constant phase element (CPE_a).

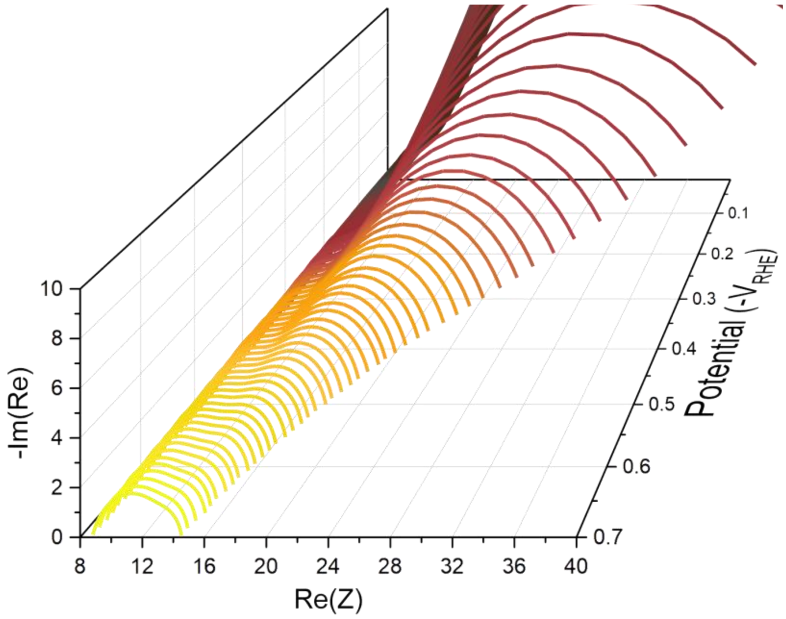

Figure 41: Example of the measured impedances for an untreated Stainless-steel electrode during the HER reaction of a 0.8 cm^2 piece in 0.1M KOH.

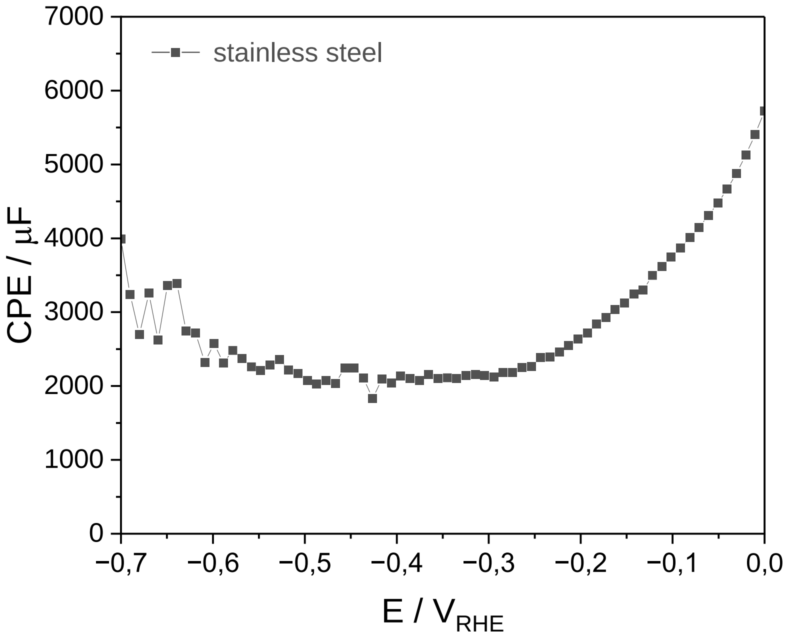

Figure 42: Plot of CPE vs. Potential of stainless steel to CPE vs. Potential to determine the optimal window to extract the adsorption capacitance value during HER reaction of a 0.8 cm^2 piece in 0.1M KOH.

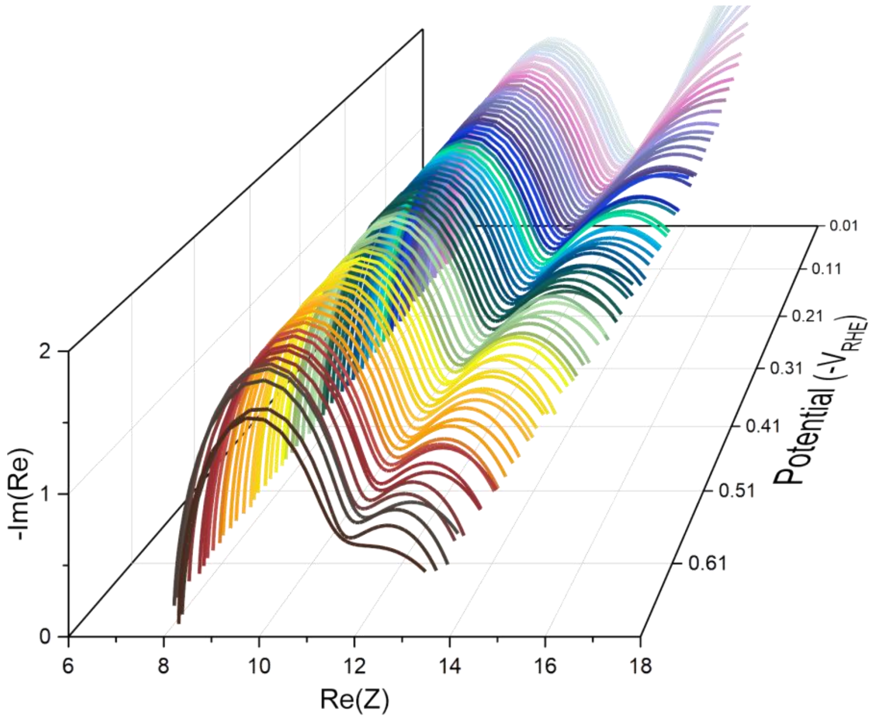

Figure 43: Impedances: of Nickel Molybdenum coated electrodes during HER of a 0.8 cm^2 piece in 0.1M KOH.

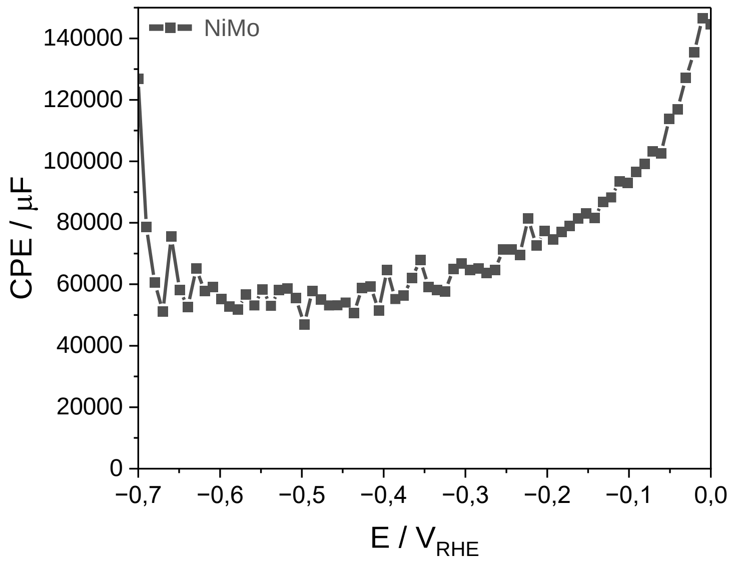

Figure 44: CPE vs. Potential to determine the optimal window to extract the adsorption capacitance value during HER of a 0.8 cm^2 piece in 0.1M KOH.

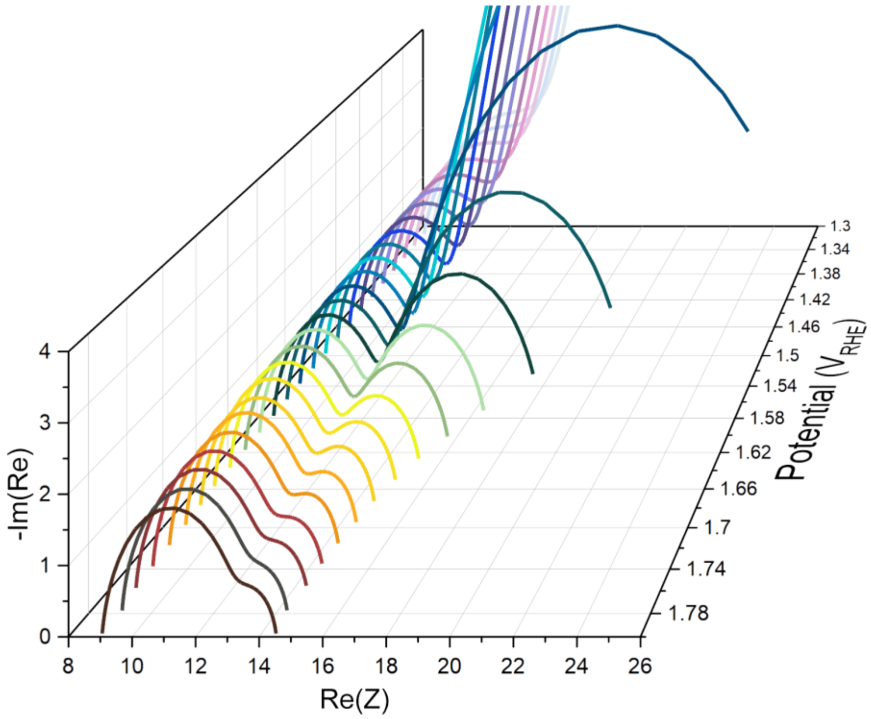

Figure 45: Fitted PEIS for bare stainless Steel OER of a 0.8 cm^2 piece in 0.1M KOH.

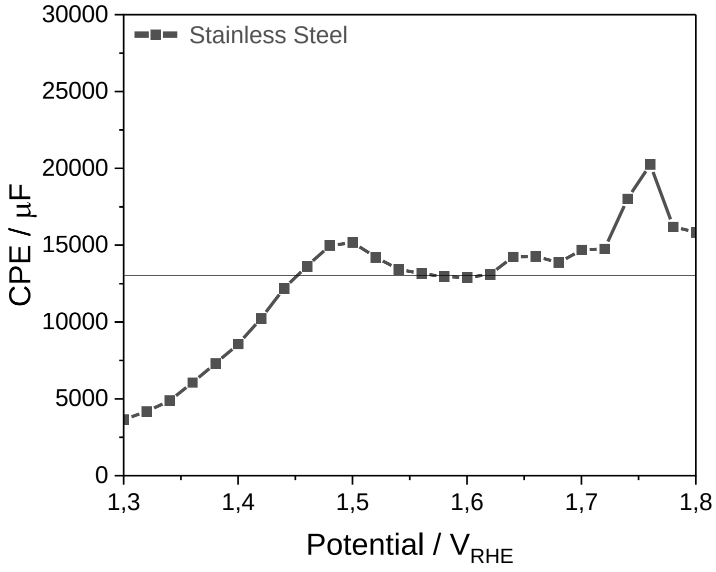

Figure 46: CPE vs. Potential to determine the optimal window to extract the adsorption capacitance value. of bare Stainless Steel for OER of a 0.8 cm^2 piece in 0.1M KOH.

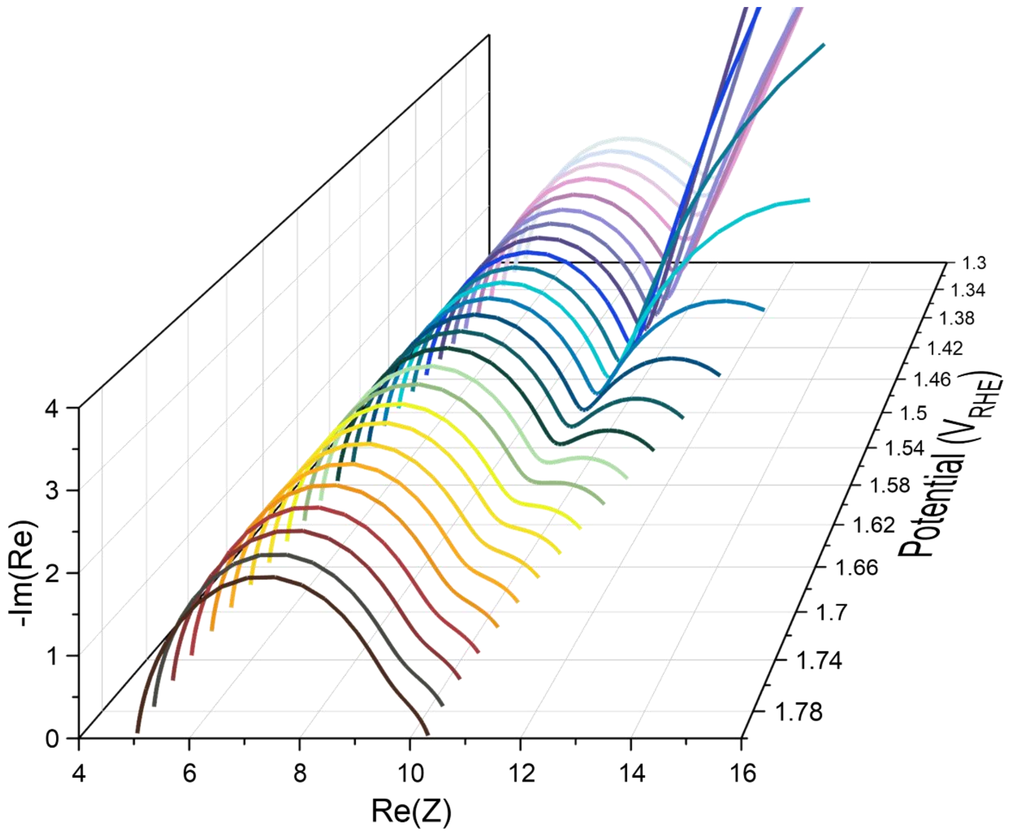

Figure 47: Fitted PEIS activated stainless Steel OER of a 0.8 cm^2 piece in 0.1M KOH.

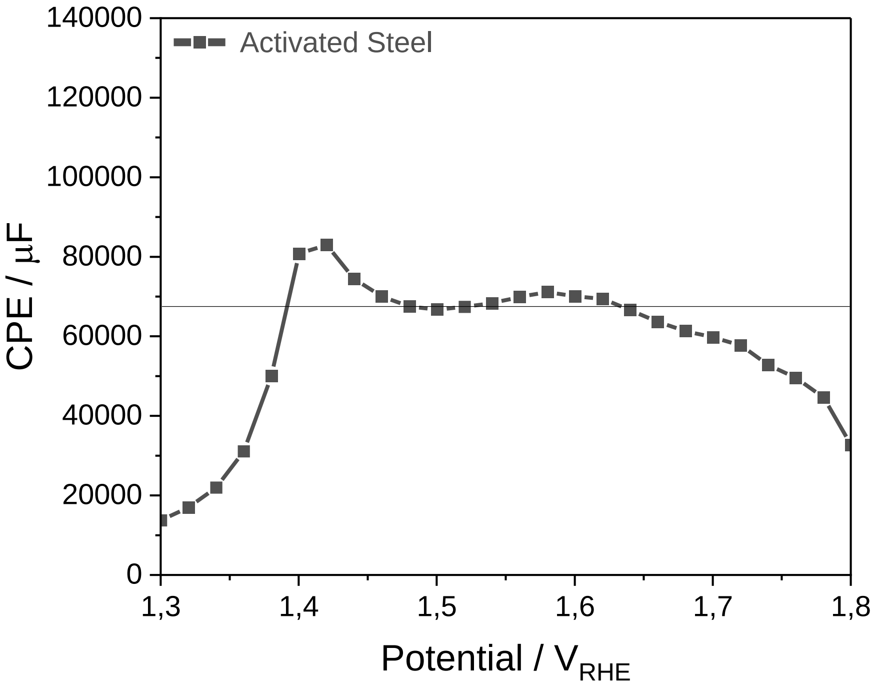

Figure 48: CPE vs. Potential to determine the optimal window to extract the adsorption capacitance value of activated steel for the OER of a 0.8 cm^2 piece in 0.1M KOH.

Table 1: ICP-MS measurements of the anolyte post AEMWE single-cell measurement.

Metal	***Concentration of metal in anolyte / mg L^{-1}***
Mo	52.9 ± 1.4
Ni	Not detectable
Fe	Not quantifiable
